# ﻿Phylogenetic placement of eight poorly known spiders of *Microdipoena* (Araneae, Mysmenidae), with descriptions of five new species

**DOI:** 10.3897/zookeys.1175.90920

**Published:** 2023-08-21

**Authors:** Qiuqiu Zhang, Yucheng Lin

**Affiliations:** 1 Key Laboratory Bio-resources and Eco-environment (Ministry of Education), College of Life Sciences, Sichuan University, Chengdu 610065, China Sichuan University Chengdu China; 2 The Sichuan Key Laboratory for Conservation Biology of Endangered Wildlife, Sichuan University, Chengdu 610064, China Sichuan University Chengdu China

**Keywords:** Description, Georgia, Indonesia, Laos, mysmenids, new record, new species

## Abstract

Ten species of the spider genus *Microdipoena* Banks, 1895 are reported from China, Laos, Indonesia, Georgia, and Seychelles. DNA sequences of the eight species are obtained to confirm their correct identification. The molecular phylogenetic analysis based on five gene fragments (16S, 18S, 28S, COI, and H3) were used to test the relationships and taxonomic placements of eight *Microdipoena* species, of which five species are documented as new to science: i.e., *M.huisun***sp. nov.** (♀, China), *M.lisu***sp. nov.** (♀, China), *M.shenyang***sp. nov.** (♂♀, China), *M.thatitou***sp. nov.** (♀, Laos), and *M.zhulin***sp. nov.** (♂♀, China). Five known species are redescribed: *M.elsae* Saaristo, 1978 (♂♀, Seychelles), *M.gongi* (Yin, Peng & Bao, 2004) (♂♀, China), *M.menglunensis* (Lin & Li, 2008) (♂♀, China), *M.jobi* (Kraus, 1967) (♂♀, Georgia), and *M.yinae* (Lin & Li, 2013) (♂♀, China). All but *M.menglunensis* are diagnosed and illustrated. The family Mysmenidae is also the first recorded from Laos and Georgia.

## ﻿Introduction

Since its inception, the genus *Microdipoena* Banks, 1895 had a very confusing taxonomic history lasting more than a century. Originally considered as a monotypic genus, it was placed in Theridiidae Sundevall, 1833. Its generotype, *Microdipoenaguttata* Banks, 1895, was designated from Long Island in New York (Banks 1895). Bishop and Crosby (1926) synonymized *Microdipoena* with *Mysmena* Simon, 1894 that was accepted by Levi (1956) and Gruia (1977). At almost the same time, *Mysmena* was transferred from Theridiidae to Symphytognathidae Hickman, 1931 by Forster (1959), and subsequently to Mysmenidae Petrunkevitch, 1928 by Forster and Platnick (1977). Saaristo (1978) considered that the male palp and epigyne of *Microdipoenaguttata* are quite different in structure and revalidated this genus that consists of two species: *M.guttata* and *M.elsae*. Shortly thereafter, Brignoli (1980) reported some Oriental and Australian mysmenids and transferred *Mysmenajobi* in Symphytognathidae (Kraus 1967), *Mysmenaillectrix* and *Mysmenasaltuensis* in Theridiidae (Simon, 1895) to his created genus *Mysmenella* Brignoli, 1980. Over the next three decades, some *Microdipoena* species were placed in so-called “*Mysmenella*” by other arachnologists (e.g., Baert 1984a, b, 1989; Ono 2007; Yin et al. 2004; Lin and Li 2008, 2013). Based on the phylogenetic hypothesis of Mysmenidae, Lopardo and Hormiga (2015) re-diagnosed and circumscribed *Microdipoena* and proposed it synonymized with *Anjouanella* Baert, 1986 and *Mysmenella* Brignoli, 1980. At this point, the placement and circumscription of this genus has been reasonably confirmed.

*Microdipoena* is distributed almost worldwide except Antarctica, although it currently consists of only 16 valid species, accounting for about 8.7 percent of 183 mysmenid species (WSC 2023). Known congeners are mainly distributed in Eurasia, Africa, Americas, some Oceanic and Pacific islands.

This paper reports our findings on the study of an inventory specimens collected from China, Laos, Indonesia, Georgia, and Seychelles during 2006 through to 2018, which revealed a total of nine *Microdipoena* species, including five new to science and four previously known species. The purpose of this study are to sequence five genes of these species, to test their phylogenetic positions and relationships within the genus *Microdipoena*, and to describe and illustrate the five new *Microdipoena* species from China and Laos. This paper is also the first report of Mysmenidae from Laos and Georgia.

## ﻿Materials and methods

### ﻿Species sampling and preservation

Specimens were collected by hand or sifting from leaf litter. All of the specimens were preserved in a 95% ethanol solution at -20 °C. All examined materials and molecular vouchers involved in this study are stored in the Natural History Museum of Sichuan University in Chengdu (**NHMSU**), China.

### ﻿Molecular data

To test taxonomic position of these novel species in this study within the Mysmenidae, twelve individuals from eight species were picked out from the examined materials for molecular sequencing. Their prosoma and legs were used to extract genomic DNA and sequence five gene fragments: 16S, 18S, 28S, COI, and H3. Primer pairs and PCR protocols are given in Table 1. The abdomens and male palps were kept as vouchers. Whole genomic DNA was extracted from tissue samples with the TIANamp MicroDNA Kit (TIANGEN) following the manufacturer’s protocol for animal tissue. The five gene fragments were amplified in 25 μL reactions. Raw sequences were edited and assembled using BioEdit v. 7.2.5 (Hall 1999). Newly obtained DNA sequence data has been uploaded to GenBank for preservation (accession numbers given in Table 2).

**Table 1.** Primers and amplification conditions used in PCR.

Locus	Annealing temperature/time	Direction	Primer	Sequence 5'→3'	Reference
16S	46.45 °C/30 s	F	16sb2_12864	CTCCGGTTTGAACTCAGATCA	Hormiga et al. 2003
	43.65 °C/30 s	R	LR-J-13360	GTAAGGCCTGCTCAATGA	In this study
18S	52.1 °C/30 s	F	18S-1F	TACCTGGTTGATCCTGCCAGTAG	Giribet et al. 1996
		R	SSU rRNA reverse	GTGGTGCCCTTCCGTCAATT	Balczun et al. 2005
28S	54.9 °C/30 s	F	28sa	GACCCGTCTTGAAACACGGA	Rix et al. 2008
		R	LSUR	GCTACTACCACCAAGATCTGCA	
COI	48.95 °C/30 s	F	LCO1490	GGTCAACAAATCATAAAGATATTGG	Folmer et al. 1994
	46 °C/30 s	R	HCO2198	TAAACTTCAGGGTGACCAAAAAATCA	
H3	50 °C/30 s	F	H3nf	ATGGCTCGTACCAAGCAGAC	Colgan et al. 1998
		R	H3nr	ATRTCCTTGGGCATGATTGTTAC	

We used these new sequences and a selection from previously sequenced taxa to assemble a partial phylogeny of mysmenid spiders, which only involved five representative genus. A total of 26 mysmenid species was used for phylogenetic analysis (Table 2). The ingroup includes 11 known, nine undescribed, and four new mysmenid species. Two *Maymena* species were used as outgroups (see gray region in Table 2). We used the MAFFT v. 7.450 online server (https://mafft.cbrc.jp/alignment/server/) with default parameters to align the sequences of the eight *Microdipoena* species. All sequences were concatenated in sequences Matrix v. 1.7.8 (Vaidya et al. 2011). We used PartitionFinder2 (Lanfear et al. 2017) to identify the best-fit models of molecular evolution for each locus. GTR+I+G was selected for COI, H3, 18S, and 28S, and GTR+G was selected for 16S.

**Table 2.** List of 26 mysmenid species and their DNA data used for molecular phyligenetic analysis (including new sequences data obtained from asterisked species* in this study).

Species	Identifier	Source	16S	18S	28S	COI	H3
*Maymenaambita*	*Maymenaambita*	NCBI	GU456746	GU456765	GU456824	GU456876	GU456921
*Maymenamayana*	*Maymenamayana*	NCBI	HM030403	HM030411	HM030421	–	–
*Troglonetagranulum*	*Troglonetagranulum*	NCBI	HM030409	HM030418	HM030429	–	–
*Troglonetayuensis*	XX52F	NHMSU	MZ612929	MZ613003	MZ613076	MZ584802	MZ603614
	XX52M	NHMSU	MZ612930	MZ613004	MZ613077	MZ584803	MZ603615
Troglonetayunnanensis*	GlgMY49F	NHMSU	OQ756232	OQ756222	OQ756224	OQ756244	OQ753870
	GlgMY49M	NHMSU	OQ756233	OQ756223	OQ756225	OQ756245	OQ753871
*Yamanetakehen*	GlgMY15F	NHMSU	MK908793	MK908809	MK908801	MK895534	MK895542
	GlgMY15M	NHMSU	MK908792	MK908808	MK908800	MK895533	MK895541
*Yamanetapaquini*	GlgMY16M	NHMSU	MK908794	MK908810	MK908802	MK895535	MK895543
	GlgMY16F	NHMSU	MK908795	MK908811	MK908803	MK895536	MK895544
*Mysmena* sp. MYSM006MAD	MYSM-006-MAD	NCBI	–	GU456774	GU456832	GU456882	GU456929
*Mysmena* sp. MYSM011ARG	MYSM-011-ARG	NCBI	–	GU456779	GU456837	–	GU456934
*Mysmena* sp. MYSM018MAD	MYSM-018-MAD	NCBI	–	GU456785	GU456843	–	GU456939
*Mysmena* sp. MYSM028MAD	MYSM-028-MAD	NCBI	–	GU456795	GU456851	GU456893	–
Mysmenidae sp._MD2476	Mysmenidae sp._MD2476	NCBI	–	–	MT651634	–	–
Mysmeninae sp._7502_050	Mysmeninae sp._7502_050	NCBI	MG947326	–	–	–	–
Mysmeninae sp._Fuzhou	Fuzhou-Dahuxiang-32	NCBI	–	–	–	MF467659	–
*Microdipoenanyungwe*	*Microdipoenanyungwe*	NCBI	GU456748	GU456767	GU456826	GU456878	GU456923
*Microdipoenaguttata*	*Microdipoenaguttata*	NCBI	GU456747	GU456766	GU456825	GU456877	GU456922
*Microdipoena* sp. AtoL_ARAGH000003	ARAGH000003	NCBI	HM030404	HM030412	HM030422	HM030432	HM030439
*Microdipoena* sp. MYSM-030-MAD	MYSM-030-MAD	NCBI	–	GU456797	–	GU456895	GU456948
Microdipoenaelsae*	SEY02F	NHMSU	OQ780298	OQ780273	OQ780288	OQ739554	OQ753867
Microdipoenagongi*	XX53F	NHMSU	OQ780302	OQ780277	OQ780292	OQ739558	OQ753861
	XX53M	NHMSU	–	OQ780278	OQ780293	OQ739559	OQ753862
*Microdipoenahuisun* sp. nov.*	TW03F	NHMSU	OQ780299	OQ780274	OQ780289	OQ739555	–
*Microdipoenalisu* sp. nov.*	GlgMY83F	NHMSU	OQ780295	OQ780268	OQ780283	OP462192	OQ753864
Microdipoenamenglunensis*	XSBN20F	NHMSU	OQ780300	OQ780275	OQ780290	OQ739556	OQ753868
	XSBN20J	NHMSU	OQ780301	OQ780276	OQ780291	OQ739557	OQ753869
*Microdipoenashenyang* sp. nov*	LN01F	NHMSU	–	OQ780269	OQ780284	–	–
	LN01M	NHMSU	–	OQ780270	OQ780285	–	–
Microdipoenayinae*	SC11F	NHMSU	OQ780296	OQ780271	OQ780286	OQ739552	OQ753866
	SC11M	NHMSU	OQ780297	OQ780272	OQ780287	OQ739553	OQ753865
*Microdipoenazhulin* sp. nov.*	GX02F	NHMSU	OQ780294	OQ780267	OQ780282	OQ739551	OQ753863

Topology The maximum likelihood (ML) tree was constructed using Phylosuite v. 1.2.2 (Zhang et al. 2020) with TBR (Tree-Bisection-Reconnection) branch swapping and 2000 bootstrap replicates with default parameters. Bayesian phylogenetic inference (BI) was performed using MrBayes v. 3.2.7 (Ronquist et al. 2012) through the Cipres Science Gateway (Miller et al. 2010) using four Markov Chain Monte Carlo (MCMCs) chains with default heating parameters for 50,000,000 generations or until the average standard deviation of split frequencies was less than 0.01. The Markov chains were sampled every 1000 generations, and the first 25% of sampled trees were burn-in. The program Tracer v. 1.7.1 (Rambaut et al. 2018) was used to analyze the performance of our BI analyses.

### ﻿Morphological data

Specimens were examined and measured using a Leica M205 C stereomicroscope. Further details were studied with an Olympus BX 43 compound microscope. Male palps and epigynes dissected from the bodies were photographed with a Canon EOS 60D wide zoom digital camera (8.5 megapixels) mounted on an Olympus BX 43 compound microscope. The individual spider was photographed directly under the compound microscope after being reshaped to its natural status. To show more detailed features, epigynes and each disassembled parts of male palps were treated with lactic acid before being embedded in Hoyer`s gum to take photos of the vulvae. The images were montaged using Helicon Focus 3.10.3 (Khmelik et al. 2006) image stacking software.

All measurements are in millimeters. Leg measurements are given as follows: total length (femur, patella, tibia, metatarsus, and tarsus). References to figures in the cited papers are in lowercase (fig. or figs), figures in this paper are noted with an initial capital (Fig. or Figs). Nomenclature of the genital structures was mainly based on Lopardo et al. (2011) for *Microdipoena*. Abbreviations of terms or institutions used in the text or figures are as follow:

**Male palp**

**CT** cymbial tooth;

**Cy** cymbium;

**CyC** cymbial conductor;

**CyF** cymbial fold;

**CyFs** setae on cymbial fold;

**CyP** cymbial process;

**E** embolus;

**Pa** patella;

**PC** paracymbium;

**SD** spermatic duct;

**T** tegulum;

**TC** tegular conductor;

**Ti** tibia.

**Epigyne**

**CD** copulatory duct;

**FD** fertilization ducts;

**S** spermathecae;

**Sp** scape.

**Somatic morphology**

**AP** abdominal protuberance;

**FS** femoral spot;

**MC** Metatarsal clasping spine;

**TS** tibial macrosetae on male leg I.

**Institutions acronyms**

**HNU** CCollege of Life Science, Hunan Normal University, Changsha, China;

**IZCAS**Institute of Zoology, Chinese Academy of Sciences, Beijing, China;

**NHMSU**Natural History Museum of Sichuan University, Chengdu, China;

**SMF**Senckenberg Research Institute, Frankfurt, Germany;

**ZMUTU**Zoological Museum, University of Turku, Turku, Finland.

## ﻿Results

### ﻿Phylogenetic analysis

**Figure 1.**
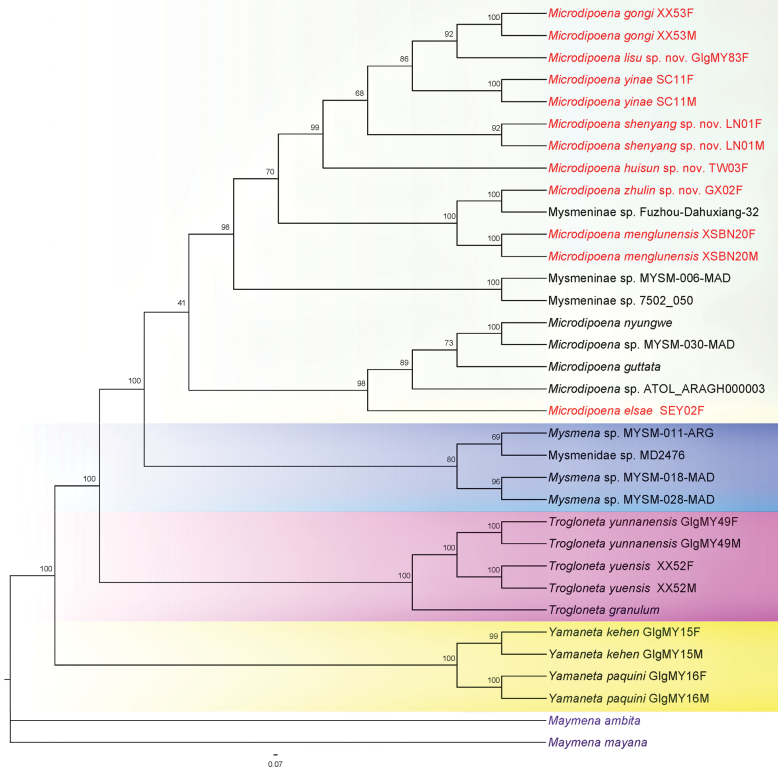
Tree topology obtained by maximum likelihood in IQ-TREE v. 2.0 using combined genes of 18 mysmenid species from NCBI plus eight *Microdipoena* species (red font). Numbers at nodes indicate bootstrap values. Note fifteen species representing the genus *Microdipoena* are clustered into a monophyly but with low support; the high support of eight species (red font) in the genus *Microdipoena* (pale box). *Mysmena* (including four undescribed species in blue box), *Trogloneta* (pink box) and *Yamaneta* (yellow box) are also monophyletic and with high support respectively.

**Figure 2.**
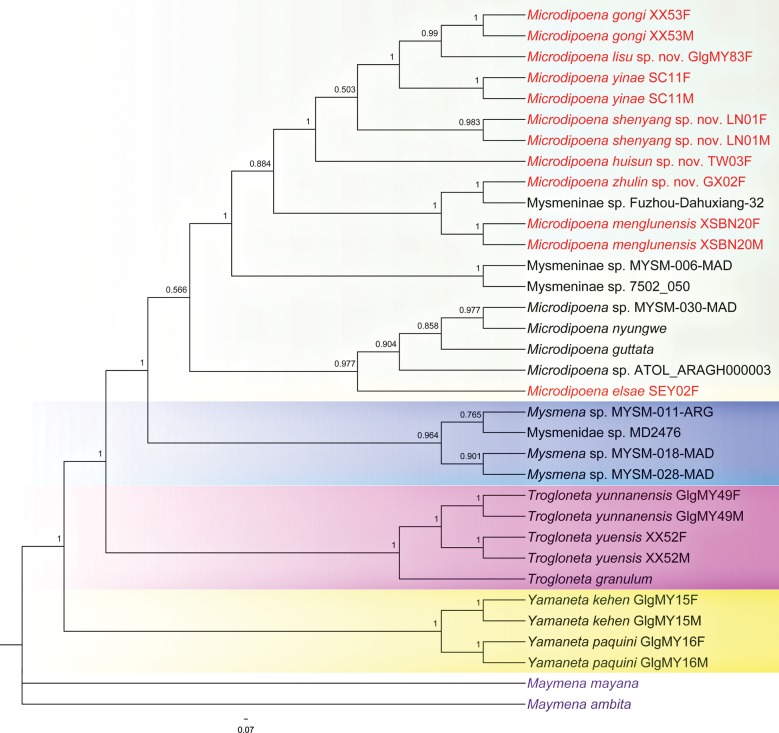
Tree topology from Bayesian analysis in MrBayes v. 3.2.7. Numerical values at nodes indicate posterior probabilities; other comments as in Fig. 1. Note the high support of eight species (red font) in the clade of *Microdipoena* (pale box), which is monophyletic but with low support. Other three clades, *Mysmena* (blue box), *Trogloneta* (pink box), and *Yamaneta* (yellow box), are also monophyletic respectively and with high support.

The topology inferred by the two different phylogenetic analyses based on the combined sequence dataset of five gene fragments performed (Figs 1, 2) show high consistencies in several mysmenid groupings. High support values are common at each end clade. Except for two *Maymena* species designated as outgroup, the remaining 24 mysmenid species are divided into four major clades, each of which represents a different genus. Both ML and BI trees analyses recovered *Microdipoena* as monophyletic and a sister group of *Mysmena* + *Trogloneta* + *Yamaneta*.

In the ML tree, the clade of *Yamaneta* represented by two known species (*Y.kehen* and *K.paquini*) is near the base of ML and BI trees, with high support (indicated by yellow box in Fig. 1). The clade of *Trogloneta* containing three known species (*T.granulum*, *T.yuensis*, and *T.yunnanensis*) is also monophyletic with high support (indicated by pink box in Fig. 1). Four undescribed *Mysmena* (MYSM-011-ARG, MYSM-018-MAD, and MYSM-028-MAD) and Mysmenidae species (Mysmenidae sp._MD2476) composed the clade of *Mysmena* shown in the blue box in Fig. 1, which is located in the middle of the topological structure trees, between the clades of *Trogloneta* and of *Microdipoena*, and also has relatively high support. A clade composed of ten *Microdipoena* species (including eight species involved in this study, indicated by red font in the pale box in Fig. 1) and five undescribed species (Fuzhou-Dahuxiang-32, MYSM-006-MAD, MYSM-030-MAD, Mysmeninae sp._7502_050, and AtoL_ARAGH000003) were monophyletic, but with low support. These results support our taxonomic classification.

The result of BI is consistent with ML for all major clades (Fig. 2). In the BI topology, seven Chinese and one Seychelles species involved in our study (indicated by red font in Fig. 2), together with two known species (*Microdipoenanyungwe*, *M.guttata*), two undescribed species (MYSM-030-MAD, AtoL_ARAGH000003), and three undescribed Mysmeninae species (Fuzhou-Dahuxiang-32, MYSM-006-MAD, and Mysmeninae sp._7502_050) form a separate monophyletic, lower supported clade compared to *Mysmena*, *Trogloneta*, and *Yamaneta*. However, each species has high support at each end clade of the BI tree respectively. The available molecular evidence seems sufficient to justify the taxonomic placement of four new and four known *Microdipoena* species in this study.

### ﻿Taxonomy

**Family Mysmenidae Petrunkevitch, 1928**

#### ﻿Genus Microdipoena

Taxon classificationAnimaliaAraneaeMysmenidae

Banks, 1895

3A367307-E9F3-5E6A-AB71-A52529288571

Mysmena Simon, 1895: 149.
Microdipoena Banks, 1895: 85 (synonymized by Bishop and Crosby 1926: 127).
Mysmena Bishop & Crosby, 1926: 177.
Microdipoena Saaristo, 1978: 124.
Mysmenella Brignoli, 1980: 731 (synonymized by Lopardo and Hormiga 2015: 783).
Anjouanella Baert, 1986: 265 (synonymized by Lopardo and Hormiga 2015: 783).
Microdipoena Lopardo & Hormiga, 2015: 783.

##### Type species.

*Microdipoenaguttata* Banks, 1895 by original designation; type locality Long Island, New York, USA.

##### Diagnosis.

**Figure 3.**
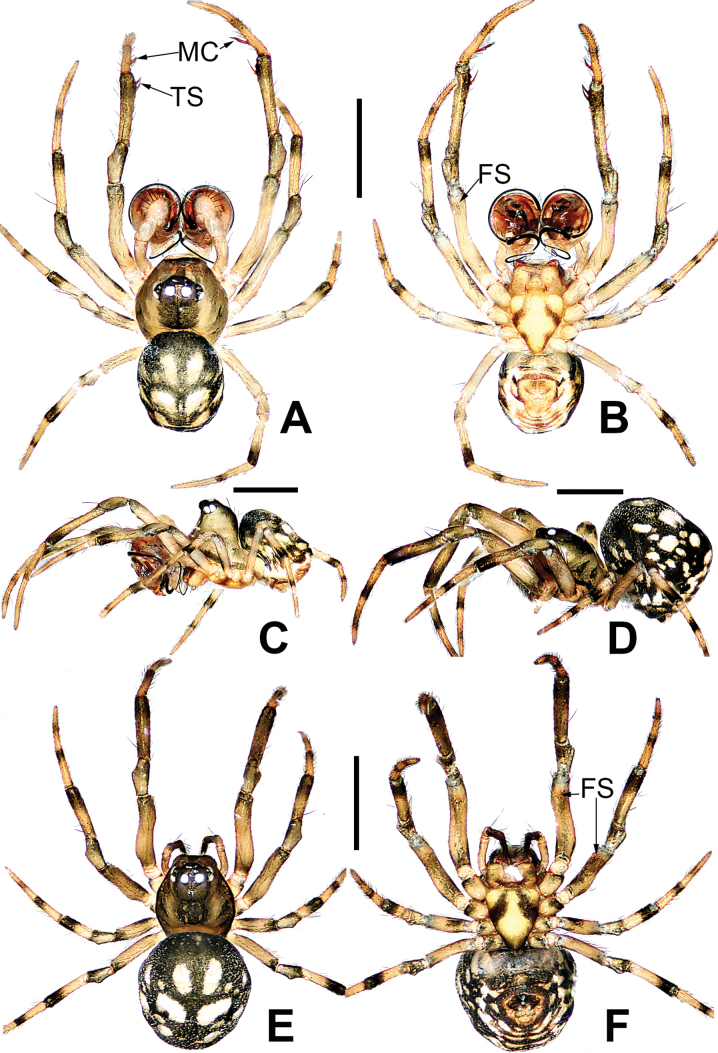
*Microdipoenaelsae* Saaristo, 1978, male **(A–C)** and female **(D–F)** from Seychelles **A** habitus, dorsal **B** habitus, ventral **C** habitus, lateral **D** habitus, lateral **E** habitus, dorsal **F** habitus, ventral. Abbrrviations: FS = femoral spot, MC = Metatarsal clasping spine, TS = tibial spine on male leg I. Scale bars: 0.50 mm.

**Figure 4.**
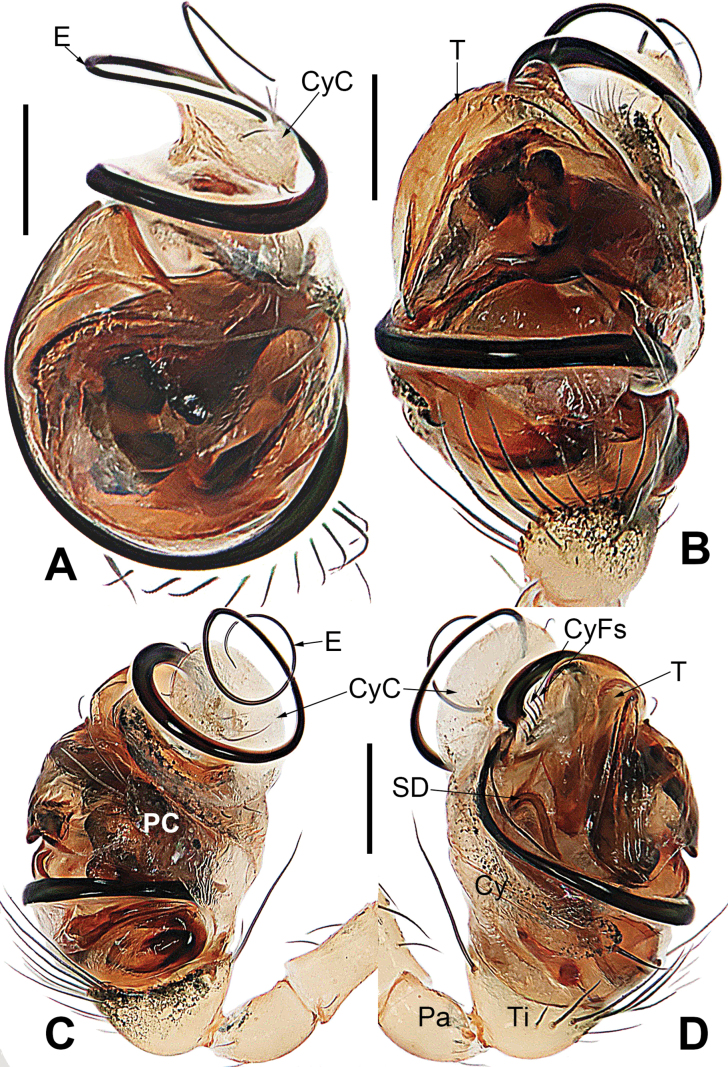
*Microdipoenaelsae* Saaristo, 1978, from Seychelles **A** male palp, apical **B** male palp, dorsal **C** male palp, prolateral **D** male palp, retrolateral. Abbreviations: Cy = cymbium, CyC = cymbial conductor, CyFs = setae on cymbial fold, E = embolus, Pa = patella, PC = paracymbium, SD = spermatic duct, T = tegulum, Ti = tibia. Scale bars: 0.10 mm.

The male can be distinguished from other mysmenids by there are two or three distal-prolateral macrosetae on the tibia I (Figs 3A, 8A, 11A, 15A, 21B). The palpal bulb is very large (at least 4–5× tibia in size, 2–3× in other mysmenids). The cymbium has an apical part with complex structure, which specialized as a cymbial conductor and a cymbial process (Figs 6H, 9C, 12B, 16B, 22D). A lobe-shaped paracymbium bears several long setae along its edge (Figs 4C, 6H, 9B, 12B, F, 16B, 20D, 22D). The thick embolus folds into a twisted complex structure at distal end, wrapped by a membranous structure on the apex of cymbium (Figs 9A–C, 12D–G, 16C–G, 20D, E, 22A, F–H) (except in *M.comorensis*, *M.elsae*, *M.guttata*, *M.mihindi*, *M.nyungwe*, and *M.vanstallei*, without complex structure, but with either a distal apophysis or irregular membrane). The female differed from other mysmenids by the abdomen with a whitish ventral ring around the spinnerets (Figs 3F, 11E, 19D, 21F) (except *M.comorensis*, with all ventral abdominal area paler). The shape of spermathecae are mostly round, oval, or semicircular, wrapped by membranous copulatory ducts from posterior or around (Figs 7C, 9F, 10F, 13C, 14F, 17C, 18F, 23D).

##### Composition.

Twenty one species: *Microdipoenacomorensis* (Baert, 1986) (♂♀), *M.elsae* Saaristo, 1978 (♂♀), *M.gongi* (Yin, Peng & Bao, 2004) (♂♀), *M.guttata* Banks, 1895 (♂♀), *M.huisun* sp. nov. (♀), *M.illectrix* (Simon, 1895) (♂), *M.jobi* (Kraus, 1967) (♂♀), *M.lisu* sp. nov. (♀), *M.menglunensis* (Lin & Li, 2008) (♂♀), *M.mihindi* (Baert, 1989) (♂), *M.nyungwe* Baert, 1989 (♂♀), *M.ogatai* (Ono, 2007) (♂♀), *M.papuana* (Baert, 1984) (♂), *M.pseudojobi* (Lin & Li, 2008) (♂♀), *M.saltuensis* (Simon, 1895) (♀), *M.samoensis* (Marples, 1955) (♂♀), *M.shenyang* sp. nov. (♂♀), *M.thatitou* sp. nov. (♀), *M.vanstallei* Baert, 1985 (♂), *M.yinae* (Lin & Li, 2013) (♂♀), and *M.zhulin* sp. nov. (♂♀).

##### Distribution.

From Europe to the Caucasus, from East Asia to Southeast Asia and South Asia to the Middle East, from central Africa to Madagascar and Seychelles, from USA to Paraguay, from New Guinea to Samoa and Hawaii.

#### ﻿ Microdipoena elsae

Taxon classificationAnimaliaAraneaeMysmenidae

Saaristo, 1978

3DCFD934-BD97-5A4E-B103-C11B266C8A30

**Figure 5.**
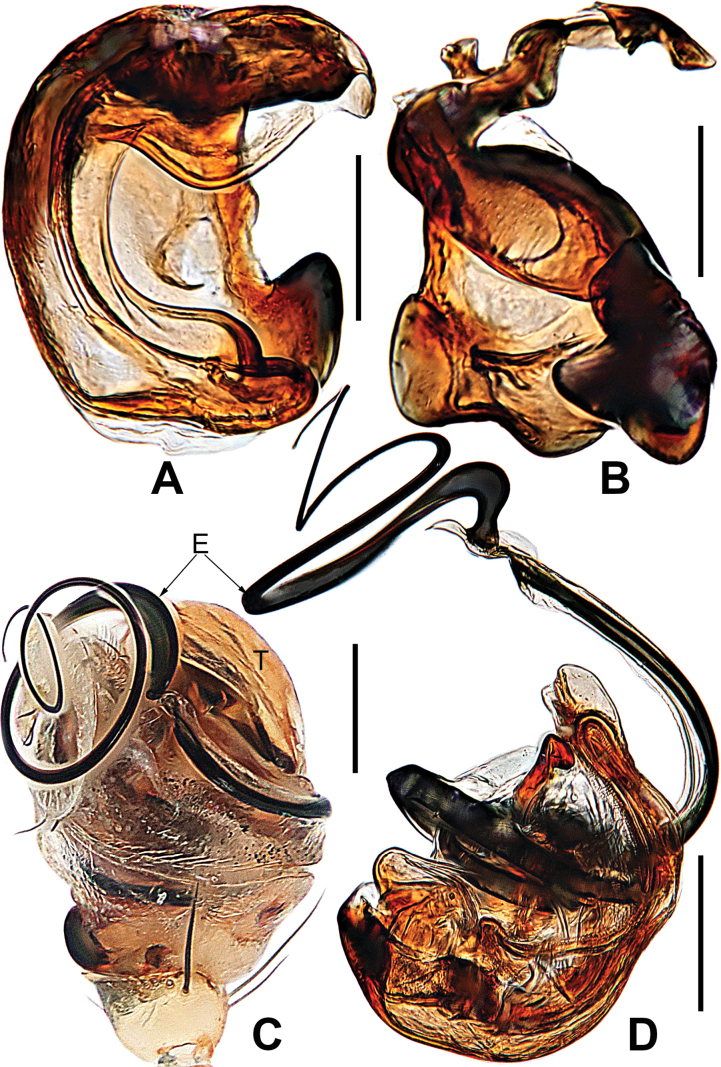
*Microdipoenaelsae* Saaristo, 1978, from Seychelles **A** conductor, from behind **B** tegulum, from behind **C** male palp, ventral **D** bulbus with conductor and tegulum removed, dextrolateral. Abbreviations: E = embolus, T = tegulum. Scale bars: 0.10 mm.

**Figure 6.**
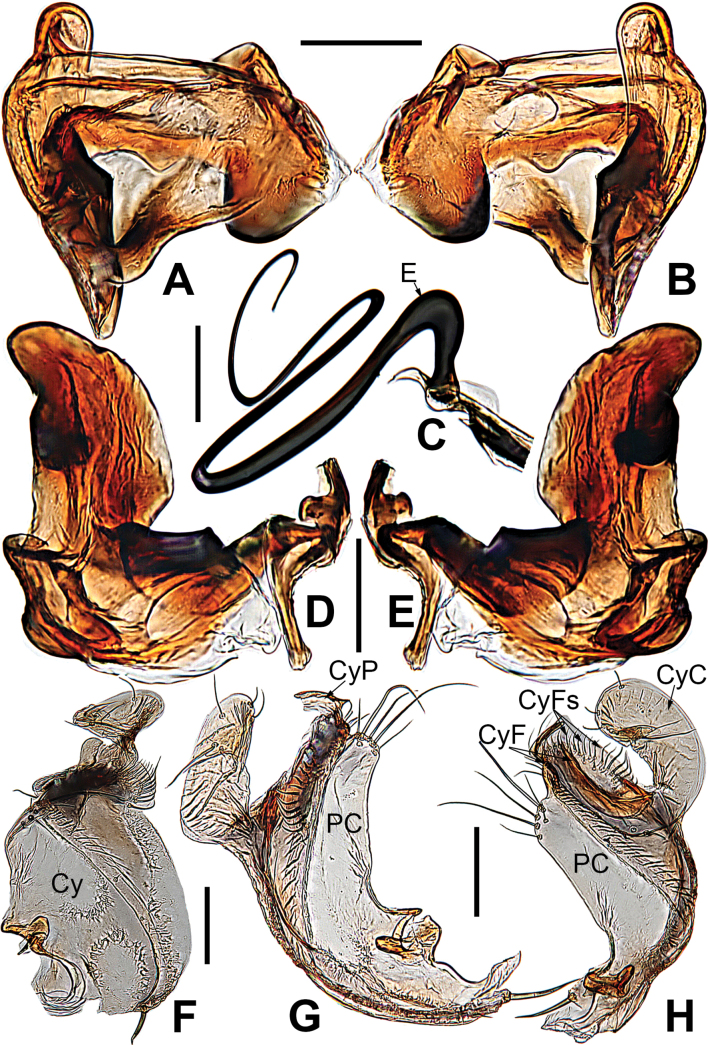
*Microdipoenaelsae* Saaristo, 1978, from Seychelles **A** conductor, dorsal **B** conductor, ventral **C** embolic distal end, dextrolateral **D** tegulum, dorsal **E** tegulum, ventral **F** cymbium, dorsal **G** cymbium, apical-lateral **H** cymbium, prolateral. Abbreviations: Cy = cymbium, CyC = cymbial conductor, CyF = cymbial fold, CyFs = setae on cymbial fold, CyP = cymbial process, PC = paracymbium. Scale bars: 0.10 mm.

**Figure 7.**
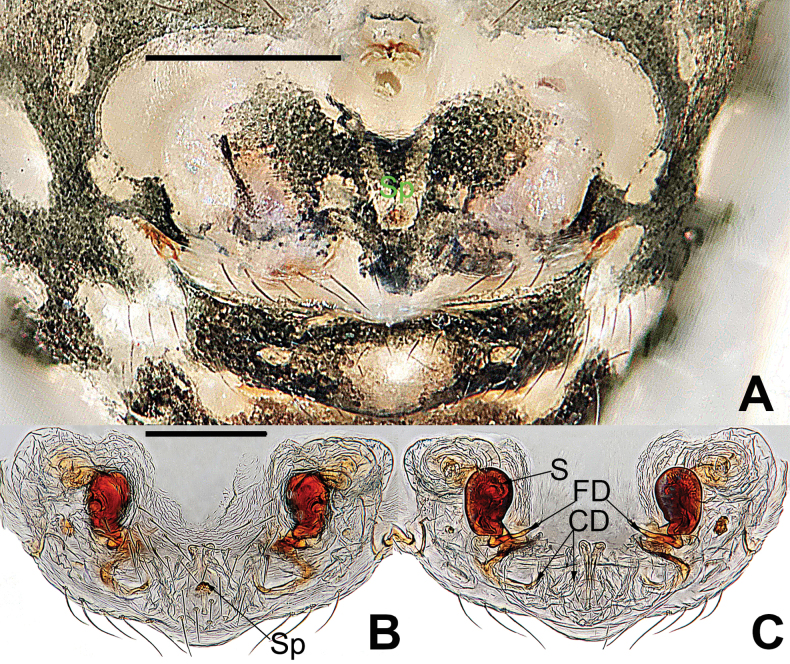
*Microdipoenaelsae* Saaristo, 1978, from Seychelles **A** epigyne, ventral **B** vulva, ventral **C** vulva, dorsal. Abbreviations: CD = copulatory duct, FD = fertilization duct, S = spermathecal, Sp = scape. Scale bars: 0.10 mm.

Figs 3 , 4 , 5 , 6 , 7

Microdipoena elsae Saaristo, 1978: 124, figs 255–265 (♂♀); Saaristo 2010: 92, fig. 17.1–17.8 (♂♀); Lopardo et al. 2011: 287, fig. 9a (♂); Lopardo and Hormiga 2015: 783, figs 17A–D, 129D, 132A, 141M, N (♂♀).
Mysmena elsae : Roberts 1978: 932, figs 65–73 (♂♀); Logunov 2022: 89, fig. 18 (♂).

##### Type material.

***Holotype*** ♀ (ZMUTU), ***allotype*** 1♀ 4♂ (ZMUTU), and ***paratypes*** 8♀ (ZMUTU) **Seychelles**: Mahé near La Misére, sieving leaf litter, 600 m elev., 30.V.1975, M. Saaristo leg. Not examined.

##### Other material examined.

2♂ 3♀ 3 juvs (NHMSU-SEY02), **Seychelles**: Mane, at half of Morne Blanc, a pile of chopped wood (4°39.553'S, 55°26.199'E; 461 m elev.), 30.VI.2013, H. Zhao leg.; 2♀ 1 juv (NHMSU-SEY01), La Digue, Belle-Vue Mountain (4°21.611'S, 55°50.470'E; 213 m elev.), 5.VII.2013, H. Zhao leg.

##### Diagnosis.

Male of *Microdipoenaelsae* differs from other congeners except for *M.comorensis* (Baert, 1986), *M.guttata* Banks, 1895, *M.nyungwe* Baert, 1989, *M.vanstallei* Baert, 1985 by the filiform embolus without a distal twisted complex structure. Its male seems to be most similar to *M.nyungwe*, but can be distinguished by the embolus having a small membranous hook at the intermediate constriction and the absence of cymbial groove (cf. Figs 5D, 6C and Lopardo and Hormiga 2015: 554, fig. 22C, F, G). Its female is similar to *M.nyungwe* and *M.guttata* but distinguished by the ovate spermathecae (round in *M.nyungwe* and *M.guttata*), the fertilization duct is slightly sclerotized and longer (cf. Fig. 7B, C vs Lopardo and Hormiga 2015: 554, 672, figs 18G, 22B, 129A, B).

##### Description.

**Male**: Total length 1.04. Carapace 0.44 long, 0.42 wide, 0.44 high. Clypeus 0.10 high. Sternum 0.33 long, 0.30 wide. Abdomen 0.60 long, 0.63 wide, 0.88 high. Length of legs: I 1.17 (0.44, 0.14, 0.25, 0.16, 0.18); II 1.06 (0.42, 0.14, 0.22, 0.12, 0.16); III 0.78 (0.22, 0.12, 0.14, 0.14, 0.16); IV 1.08 (0.25, 0.12, 0.27, 0.20, 0.24).

##### Somatic characters

**(Fig. 3A–C). *Coloration***: carapace dark brown centrally, yellow brown marginally. Ocular base black. Chelicera, endites, and labium yellow. Sternum yellow with two brown stripes. Legs yellow and black. Abdomen dark brown with white spots dorsally, yellow with brown spots ventrally. ***Prosoma***: carapace nearly pear-shaped in dorsal view and peak-shaped in lateral view. Cephalic area upheaved. Sternum triangular, slightly plump, covered with sparse, short setae. ***Legs***: covered with setae. Mating clasper on metatarsus I, two macrosetae on tibia I, femur I with femoral spot. ***Abdomen***: nearly ovoid.

***Palp*** (Figs 4–6): large, ca as big as ½ size of the carapace. Cymbium translucent, distal end specialized as a broad, collared cymbial conductor, and a small cymbial process, modified by weakly sclerotized folds and a row of stiff short setae (Figs 4D, 5C, 6F–H). Paracymbium smooth, with long setae at the edge. Conductor wide, sclerotized, with two upper and a lower processes (Fig. 6A, B). Tegulum sclerotized, with two upper (a wide, a narrow) and one lower (a narrow) processes (Fig. 6D, E). Embolus filiform, with a membranous hook at the constriction near the middle (Figs 5D, 6C), its distal part coiled into 2.5 loops around cymbial conductor (Figs 4, 5C). Spermatic ducts faintly visible through the surface of palpal bulb and cymbium.

**Female.** Total length 0.92. Carapace 0.40 long, 0.46 wide, 0.58 high. Clypeus 0.08 high. Sternum 0.28 long, 0.30 wide. Abdomen 0.52 long, 0.46 wide, 0.62 high. Length of legs: I 1.77 (0.52, 0.16, 0.40, 0.34, 0.35); II 1.38 (0.46, 0.14, 0.34, 0.20, 0.24); III 0.98 (0.30, 0.12, 0.20, 0.16, 0.20); IV 1.08 (0.30, 0.10, 0.24, 0.20, 0.24).

##### Somatic characters

**(Fig. 3D–F). *Coloration***: carapace dark brown centrally, yellow-brown marginally. Ocular base black. Chelicera, endites, and labium yellow. Sternum yellow with two brown stripes. Legs yellow and black. Abdomen dark brown with white spots dorsally, yellow with brown spots ventrally. ***Prosoma***: carapace nearly pear-shaped in dorsal view. Cephalic part slightly elevated. Sternum triangular, covered with sparse short setae. ***Legs***: covered with setae and bristles. Femurs I and II with femoral spot. ***Abdomen***: nearly globose.

***Epigyne*** (Fig. 7A–C): spermathecae heavily sclerotized, nearly vertically ovoid, spaced by ca 3× their width. Copulatory duct almost all membranous cystic structure with irregular folds, surround the entirely spermathecae, which enters the spermathecae from posteromedially after gradually harden at the posterior area of spermathecae. Weakly sclerotized fertilization duct starts at the posterolateral side of spermatheca, and then folds back toward the center of vulva (Fig. 7C).

##### Distribution.

Seychelles, Congo, and Comoros.

#### ﻿ Microdipoena gongi

Taxon classificationAnimaliaAraneaeMysmenidae

(Yin, Peng & Bao, 2004)

6123AD81-CFE2-556D-BF6A-779A47F9499F

Figs 8 , 9

Mysmenella gongi Yin, Peng & Bao, 2004: 80, figs 1–8 (♂♀).
Microdipoena gongi : Lopardo and Hormiga 2015: 783.

##### Type material.

***Holotype*** ♀ (HNU) and ***paratypes*** 1♂ 3♀ (HNU), **China**: Hunan Province, Daoxian County, Timber Mill (25°31.000'N, 111°33.000'E), 8.IV.1988; 1♀ 7♂, **China**: Hunan Province, Daoxian County, Shuangqiao Town, 1.VI.1987, L. Gong leg. Examined in 2008.

##### Other material examined.

1♂ 2♀ (NHMSU-XX53), **China**: Hunan Province, Changsha City, Yuelu District, Yuelu Mountain scenic spot, leaf litter (28°10.502'N, 112°56.391'E; 121 m elev.), 20.IV.2018, G. Zhou leg.

##### Diagnosis.

Male of *Microdipoenagongi* can be distinguished from other congeners except for *M.illectrix*, *M.jobi*, *M.menglunensis*, *M.mihindi*, *M.ogatai*, *M.papuana*, *M.pseudojobi*, *M.samoensis*, *M.shenyang* sp. nov., *M.yinae*, and *M.zhulin* sp. nov. by the embolic end twisted into a complex structure (Figs 9A, B, 12D, 16E, 20B, 22A). It can be distinguished from these aforementioned species by the cymbium wraps the bulb prolaterally or lacking a cymbial tooth (cf. Figs 9A, B, 12B, 16B, 22D; Lin and Li 2013: CP shown in fig. 21F; Lopardo and Hormiga 2015: CyP shown in fig. 132C–F). Female of *Microdipoenagongi* differs from other congeners except for *M.huisun* sp. nov., *M.ogatai*, and *M.yinae* in lacking a long, soft, membranous scape, but having a small, weakly sclerotized, small scape (cf. Figs 9F, 10F, 19H, and Ono 2007: figs 69, 70; Figs 13C, 14F, 17C, 18F, 23D and Lopardo and Hormiga 2015: fig. 129E, F), but can be distinguished from these aforementioned species by the horizontal ovate spermathecae, which is almost as large as the posterior sclerotized area of copulatory ducts (globose in *M.huisun* sp. nov., smaller spermatheca in *M.ogatai*, inclined hemispheric spermatheca in *M.yinae*) (cf. Figs 9F, 10F, 19H and Ono 2007: fig. 70).

##### Description.

**Male**: Total length 0.98. Carapace 0.44 long, 0.48 wide, 0.52 high. Clypeus 0.08 high. Sternum 0.30 long, 0.30 wide. Abdomen 0.54 long, 0.56 wide, 0.60 high. Length of legs: I 1.33 (0.48, 0.12, 0.27, 0.22, 0.24); II 1.20 (0.42, 0.12, 0.24, 0.18, 0.24); III 0.81 (0.25, 0.10, 0.16, 0.14, 0.16); IV 1.04 (0.28, 0.12, 0.24, 0.18, 0.22).

##### Somatic characters

**Figure 8.**
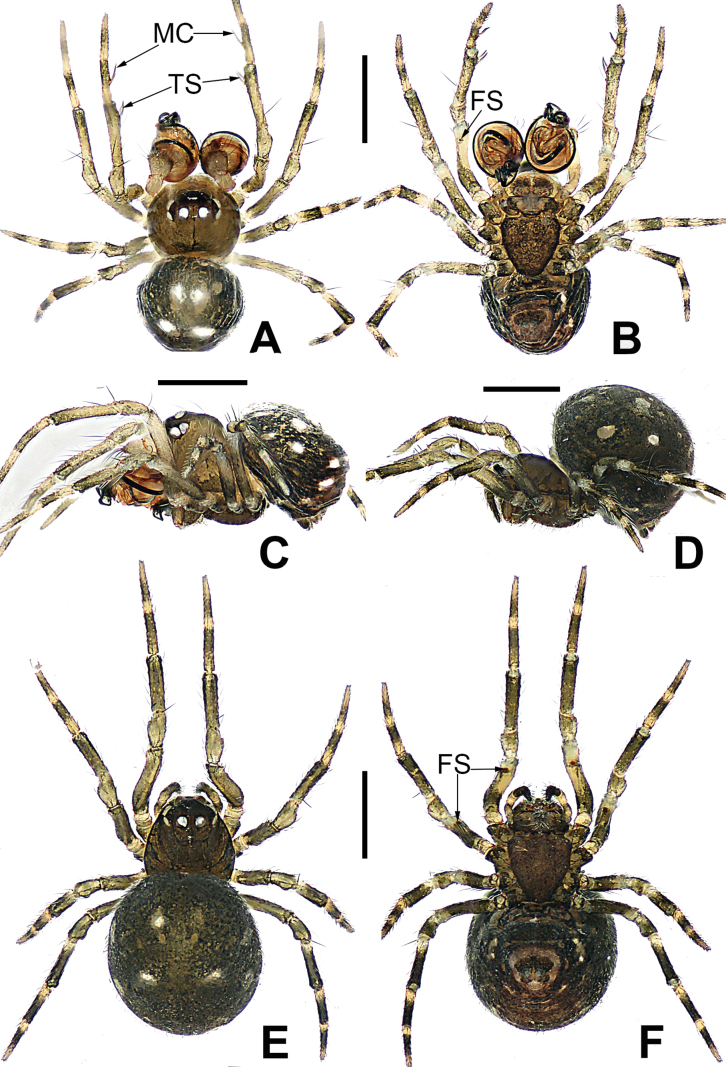
*Microdipoenagongi* Yin, Peng & Bao, 2004, male **(A–C)** and female **(D–F)**, from Hunan of China **A** habitus, dorsal **B** habitus, ventral **C** habitus, lateral **D** habitus, lateral **E** habitus, dorsal **F** habitus, ventral. Abbreviations: FS = femoral spot, MC = Metatarsal clasping spine, TS = tibial spine on male leg I. Scale bars: 0.50 mm.

**(Fig. 8A–C). *Coloration***: carapace dark brown. Ocular base black. Chelicera, endites, labium and sternum dark brown. Legs brown-black. Abdomen dark with three white spots dorsally. ***Prosoma***: carapace nearly round in dorsal view and peak-shaped in lateral view. Cephalic part flat, slightly elevated. Sternum scutiform, slightly plump. ***Legs***: covered with setae. Mating clasper on metatarsus I, two strong spines on tibia I, femur I with sclerotized femoral spot. ***Abdomen***: nearly globose.

**Figure 9.**
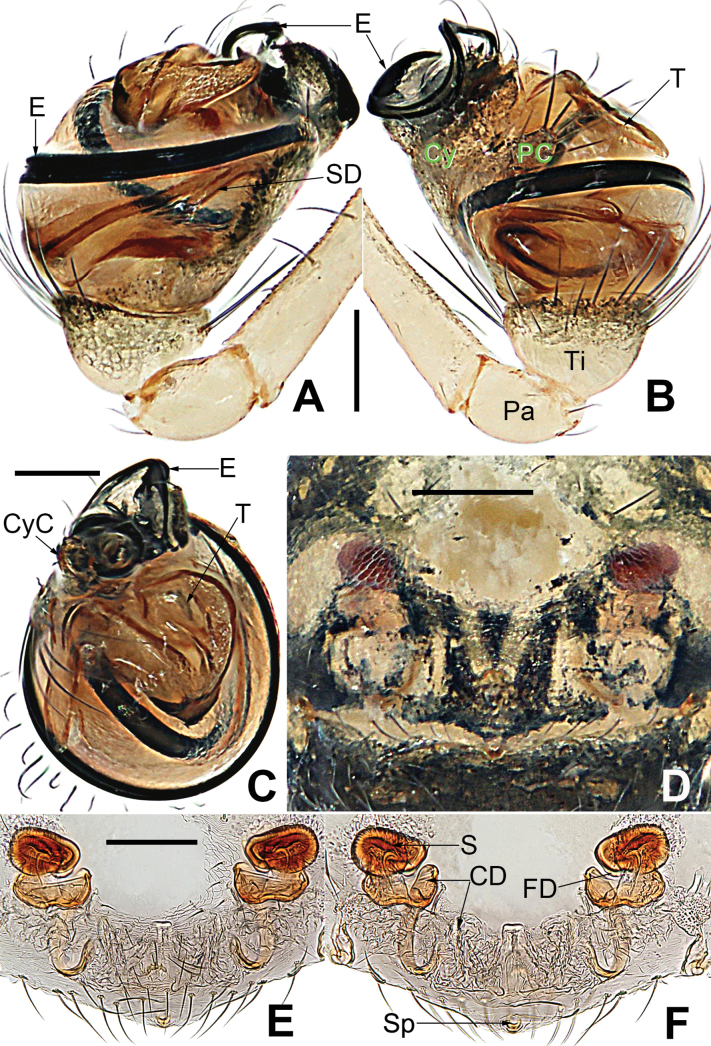
*Microdipoenagongi* Yin, Peng & Bao, 2004, from Hunan of China **A** male palp, prolateral **B** male palp, retrolateral **C** male palp, apical **D** epigyne, ventral **E** vulva, ventral **F** vulva, dorsal. Abbreviations: CD = copulatory duct, Cy = cymbium, CyC = cymbial conductor, E = embolus, Pa = patella, PC = paracymbium, S = spermathecal, SD = spermatic duct, Sp = Scape, T = tegulum, Ti = tibia. Scale bars: 0.10 mm.

***Palp*** (Fig. 9A–C): The palp 45° inclined to the surface of the tibia. Cymbium translucent, originating prolaterally, with a large cymbial conductor. Paracymbium large, finger-like, with long setae. Tegulum translucent, surface swollen. Embolus thin and relatively short, coiled into one loop over the cymbium, tip with complex structure. Spermatic ducts can be seen through translucent tegulum.

**Female.** Total length 1.37. Carapace 0.45 long, 0.52 wide, 0.40 high. Clypeus 0.10 high. Sternum 0.38 long, 0.32 wide. Abdomen 0.92 long, 0.88 wide, 0.86 high. Length of legs: I 1.32 (0.34, 0.14, 0.32, 0.28, 0.24); II 1.06 (0.22, 0.12, 0.30, 0.24, 0.18); III 0.82 (0.20, 0.12, 0.20, 0.16, 0.14); IV 0.92 (0.22, 0.12, 0.22, 0.20, 0.16).

##### Somatic characters

**(Fig. 8D–F). *Coloration***: carapace nearly black. Ocular base black. Chelicera, endites, labium black, sternum dark brown. Legs brown-black. Abdomen black with four white spots dorsally. ***Prosoma***: carapace nearly round in dorsal view. Cephalic part unraised. Sternum scutiform, slightly plump, covered with sparse setae. ***Legs***: covered with setae and bristles. Femurs I and II with sclerotized femoral spot. ***Abdomen***: nearly spherical, covered with short setae.

***Epigyne*** (Fig. 9D–F): sclerotized, length twice the width, structure can be seen slightly through the cuticle. Scape nodular shape, very small. Copulatory duct membranous, coiled under the spermathecae. Paired spermathecae transverse oval, separated by nearly double diameter. Fertilization ducts membranous, inconspicuous.

##### Distribution.

China (Hunan).

#### ﻿ Microdipoena huisun sp. nov.

Taxon classificationAnimaliaAraneaeMysmenidae

A487D059-B95B-544B-87F7-441D11C2E323

https://zoobank.org/36513469-CD9E-483A-B226-E13FBD5FC82B

Fig. 10

##### Type material.

***Holotype*** ♀ and ***paratype*** 4♀ (NHMSU-TW03), **China**: Taiwan Province, Nantou County, Huisun Forest Farm (24°05.279'N, 121°02.078'E; 788 m elev.), 1.VII.2013, G. Zheng leg.

##### Etymology.

The specific name is derived from the type locality; noun in apposition.

##### Diagnosis.

**Figure 10.**
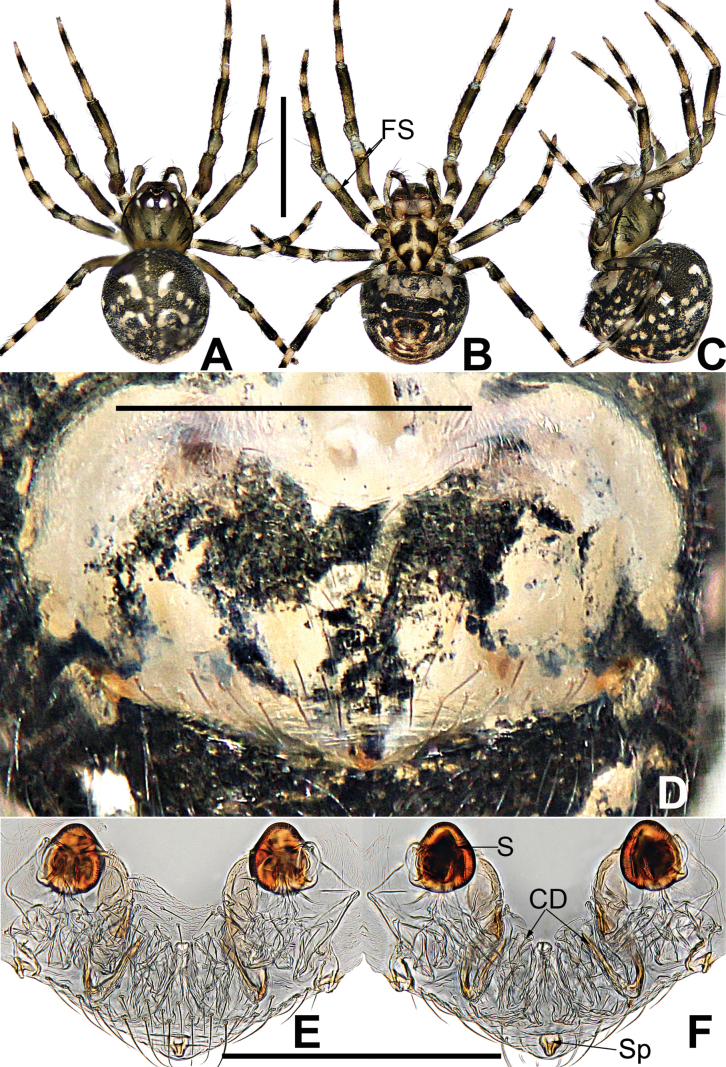
*Microdipoenahuisun* sp. nov., female from Taiwan of China **A** habitus, dorsal **B** habitus, ventral **C** habitus, lateral **D** epigyne, ventral **E** vulva, ventral **F** vulva, dorsal. Abbreviations: CD = copulatory duct, S = spermathecal, Sp = Scape. Scale bars: 0.50 mm (**A–C**), 0.10 mm (**D–F**).

This new species differs from other species except for *M.gongi*, *M.ogatai*, and *M.yinae* by the nearly spherical spermathecae and having a small scape rather than a long, soft, membranous one (cf. Figs 10E, F, 13C, 14F, 17C). It can be distinguished from *M.gongi*, *M.ogatai*, and *M.yinae* by the nearly spherical spermathecae and the narrower posterior sclerotized area of copulatory ducts (cf. Figs 10F, 9F, 19H and Ono 2007: fig. 70).

##### Description.

**Female (holotype)**: Total length 0.76. Carapace 0.28 long, 0.29 wide, 0.28 high. Clypeus 0.07 high. Sternum 0.24 long, 0.19 wide. Abdomen 0.48 long, 0.48 wide, 0.50 high. Length of legs: I 0.94 (0.30, 0.08, 0.22, 0.16, 0.18); II 0.92 (0.32, 0.08, 0.22, 0.14, 0.16); III 0.70 (0.22, 0.08, 0.14, 0.12, 0.14); IV 0.94 (0.24, 0.08, 0.24, 0.18, 0.20).

##### Somatic characters

**(Fig. 10A–C). *Coloration***: carapace nearly silver-brown centrally, black marginally. Ocular base black. Chelicera, endites yellow, labium silver-brown, sternum pale yellow with four symmetrical black spots. Legs brown-black. Abdomen black with multiple symmetrical white spots dorsally, black with symmetrical white and yellow spots ventrally. ***Prosoma***: carapace nearly pear-shaped in dorsal view. Cephalic part unraised. Sternum triangular, covered with sparse setae. ***Legs***: covered with setae and bristles. Femurs I and II with sclerotized femoral spot. ***Abdomen***: nearly oval in dorsal view, covered with sparse setae.

***Epigyne*** (Fig. 10D–F): scape nodular shape, very small. Copulatory duct membranous, coiled under the spermathecae. Paired spermathecae nearly globose, separated by nearly 3× its diameter. Fertilization ducts membranous, inconspicuous.

**Male.** Unknown.

##### Distribution.

China (Taiwan).

#### ﻿ Microdipoena jobi

Taxon classificationAnimaliaAraneaeMysmenidae

(Kraus, 1967)

218E18DF-0924-5008-9B34-67190C766FF1

Figs 11 , 12 , 13

Mysmena jobi Kraus, 1967: 392, figs 12–18 (♂♀).
Mysmenella jobi : Brignoli 1980: 731; Namkung 2002: 146, fig. 16a, b (♂♀).
Microdipoena jobi : Lopardo and Hormiga 2015: 783 (♂♀); Naumova et al. 2017: 478, figs 12–14 (♂).

##### Type material.

***Holotype*** ♂ (SMF 12958) and ***paratype*** 1♂ 2♀ (SMF 12959); 1♂ (Mus. Paris) **France**: Paris, Mainz-Gonsenheim, Gonsenheimer forest, forests trap, 20.IV.1967, W. Job leg. Examined in 2008.

##### Other material examined.

1♂ 1♀ (NHMSU-No.57), **Georgia**: Adjara, road along Acharistsqali River, dry stone debris near bridge (41°34.720'N, 41°51.760'E; 113 m elev.), 21.VII.2012, S. Li leg.

##### Diagnosis.

This species is similar to *M.gongi*, *M.illectrix*, *M.menglunensis*, *M.mihindi*, *M.ogatai*, *M.papuana*, *M.pseudojobi*, *M.samoensis*, *M.shenyang* sp. nov., *M.yinae*, and *M.zhulin* sp. nov. in having a twisted complex structure at the embolus end, but can be distinguished by the peculiar shape of conductor with a huge lower lateral process, the cymbial tooth near the ventral center of cymbium, the spherical spermathecae spaced by ca 2.3× their diameter, and the long, soft scape (cf. Figs 12B, C, F, G, 13B, C, 14F, 16A, B, 17C, 20C, 22A, B, D, 23D, and Lin and Li 2013: fig. 21F; Lin and Li 2008: figs 11F, G, 12F; Lopardo and Hormiga 2015: figs 129E, F, 132C–E).

**Figure 11.**
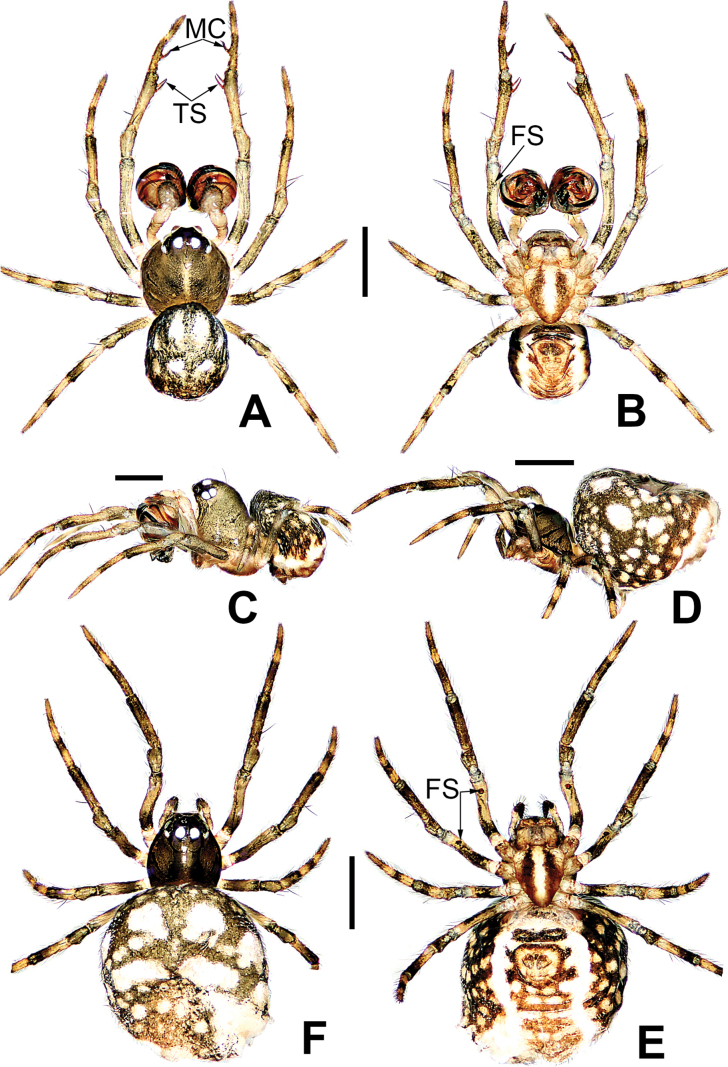
*Microdipoenajobi* Kraus, 1967, male **(A–C)** and female **(D–F)** from Georgia **A** habitus, dorsal **B** habitus, ventral **C** habitus, lateral **D** habitus, lateral **E** habitus, dorsal **F** habitus, ventral. Abbreviations: FS = femoral spot, MC = Metatarsal clasping spine, TS = tibial spine on male leg I. Scale bars: 0.50 mm.

**Figure 12.**
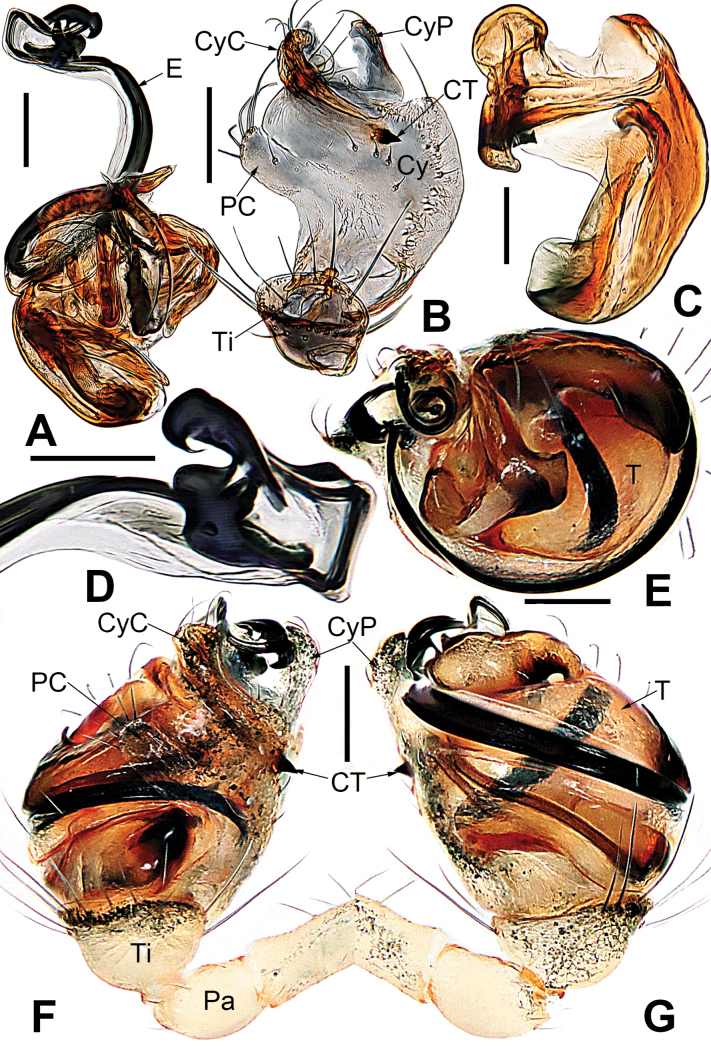
*Microdipoenajobi* Kraus, 1967 from Georgia **A** bulbus with conductor removed, from behind **B** cymbium, prolateral **C** conductor, dorsal **D** embolic end, retrolateral **E** male palp, apical **F** male palp, prolateral **G** male palp, retrolateral. Abbreviations: CT = cymbial tooth, Cy = cymbium, CyC = cymbial conductor, CyP = cymbial process, E = embolus, Pa = patella, PC = paracymbium, T = tegulum, Ti = tibia. Scale bars: 0.10 mm.

**Figure 13.**
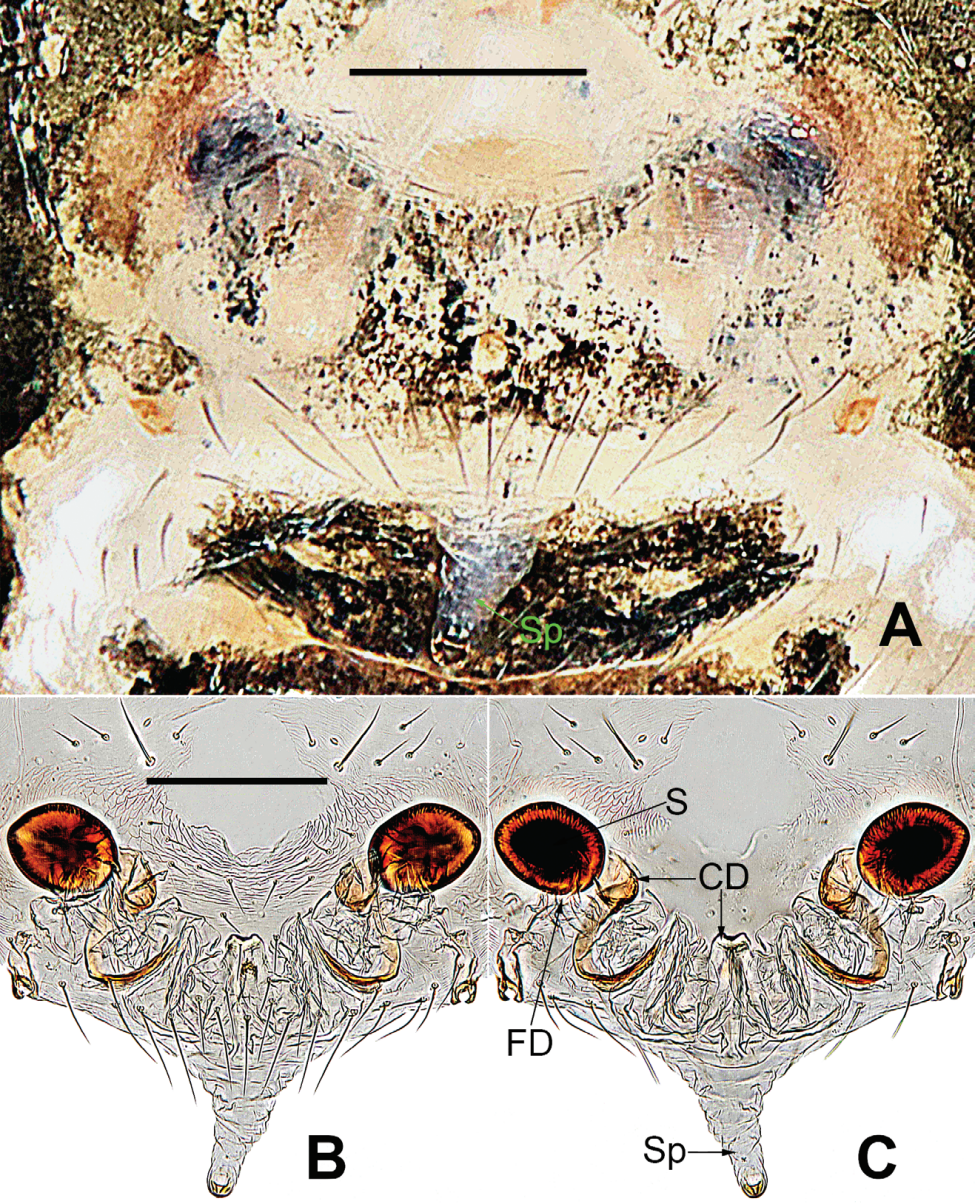
*Microdipoenajobi* Kraus, 1967 from Georgia **A** epigyne, ventral **B** vulva, ventral **C** vulva, dorsal. Abbreviations: CD = copulatory duct, FD = fertilization duct, S = spermathecal, Sp = Scape. Scale bars: 0.10 mm.

##### Description.

**Male (holotype)**: Total length 0.96. Carapace 0.46 long, 0.50 wide, 0.50 high. Clypeus 0.06 high. Sternum 0.32 long, 0.26 wide. Abdomen 0.5 long, 0.46 wide, 0.50 high. Length of legs: I 1.42 (0.52, 0.08, 0.40, 0.20, 0.22); II 1.22 (0.40, 0.08, 0.32, 0.20, 0.22); III 0.72 (0.20, 0.08, 0.18, 0.12, 0.14); IV 1.98 (0.20, 0.08, 0.26, 0.20, 0.24).

##### Somatic characters

**(Fig. 11A–C). *Coloration***: carapace silver-brown. Ocular base black. Chelicera, endites, labium yellow; sternum yellow with two longitudinal brown stripes. Legs yellow-brown. Abdomen black with symmetrical white spots dorsally, yellow with two symmetrical white stripes ventrally. ***Prosoma***: carapace nearly oval in dorsal view and peak-shaped in lateral view. Cephalic part elevated. Sternum scutiform, plump, covered with sparse setae. ***Legs***: covered with setae. Mating clasper on metatarsus I, two strong spines on tibia I, femur I with sclerotized femoral spot. ***Abdomen***: nearly oval in dorsal view.

***Palp*** (Fig. 12A–G): The palp 45° inclined to the surface of the tibia. Cymbium translucent, cymbial tooth on the prolateral, sclerotized; the tip specialized as cymbial conductor; a large cymbial process at the contralateral of the cymbial conductor. Paracymbium small, with long setae. Conductor 7-shaped, sclerotized, with two large apophyses apically and two pointed apophyses basally. Tegulum translucent, swollen surface. Embolus wide and long, the tip with complex structure, most structure coiled into two loops. Spermatic ducts can be seen through translucent tegulum.

**Female.** Total length 1.38. Carapace 0.40 long, 0.40 wide, 0.33 high. Clypeus 0.06 high. Sternum 0.32 long, 0.30 wide. Abdomen 0.98 long, 0.92 wide, 0.66 high. Length of legs: I 1.32 (0.36, 0.16, 0.30, 0.24, 0.26); II 1.12 (0.32, 0.16, 0.26, 0.18, 0.20); III 0.70 (0.16, 0.12, 0.14, 0.12, 0.16); IV 0.84 (0.32, 0.12, 0.16, 0.12, 0.12).

##### Somatic characters

**(Fig. 11D–F). *Coloration***: carapace dark brown. Ocular base black. Chelicera, endites, labium yellow; sternum yellow with two longitudinal brown stripes. Legs brown-black. Abdomen silvery brown with multiple white spots dorsally, brown with two symmetrical white stripes and multiple little white spots ventrally. ***Prosoma***: carapace nearly pear-shaped in dorsal view. Cephalic part unelevated. Sternum scutiform, slightly plump, covered with sparse setae. ***Legs***: covered with setae and bristles. Femurs I and II with sclerotized femoral spot. ***Abdomen***: nearly spherical in dorsal view, covered with sparse setae.

***Epigyne*** (Fig. 13A–C): slightly sclerotized. Scape long, base wide, with thin folds. Copulatory duct long, membranous, with sclerotized S-shaped, coiled under the spermathecae. Paired spermathecae transverse ovoid, separated by more than 3× diameter. Fertilization ducts membranous, inconspicuous.

##### Distribution.

France, Georgia (Adjara), Caucasus, Iran, China, Korea, and Japan.

#### ﻿ Microdipoena lisu sp. nov.

Taxon classificationAnimaliaAraneaeMysmenidae

26B27C2F-0485-5888-A238-27DF2B7D9764

https://zoobank.org/9BFA1810-CC60-4F33-8555-12EC6BB10603

Fig. 14

##### Type material.

***Holotype*** ♀ and ***paratype*** 1♀ (NHMSU-Glg83), **China**: Yunnan Prov., Gongshan Co., Bingzhongluo Town, halfway from Bingzhongluo to Puhuasi Temple, leaf litter in roadside native forest (28°01.420'N, 098°36.133'E, 1867 m elev.), 15.VIII.2018, Y. Lin et al. leg.

##### Etymology.

The new species is named after the Lisu people, an ethnic minority mainly living in the Nujiang River basin in Yunnan Prov.; noun in apposition.

##### Diagnosis.

**Figure 14.**
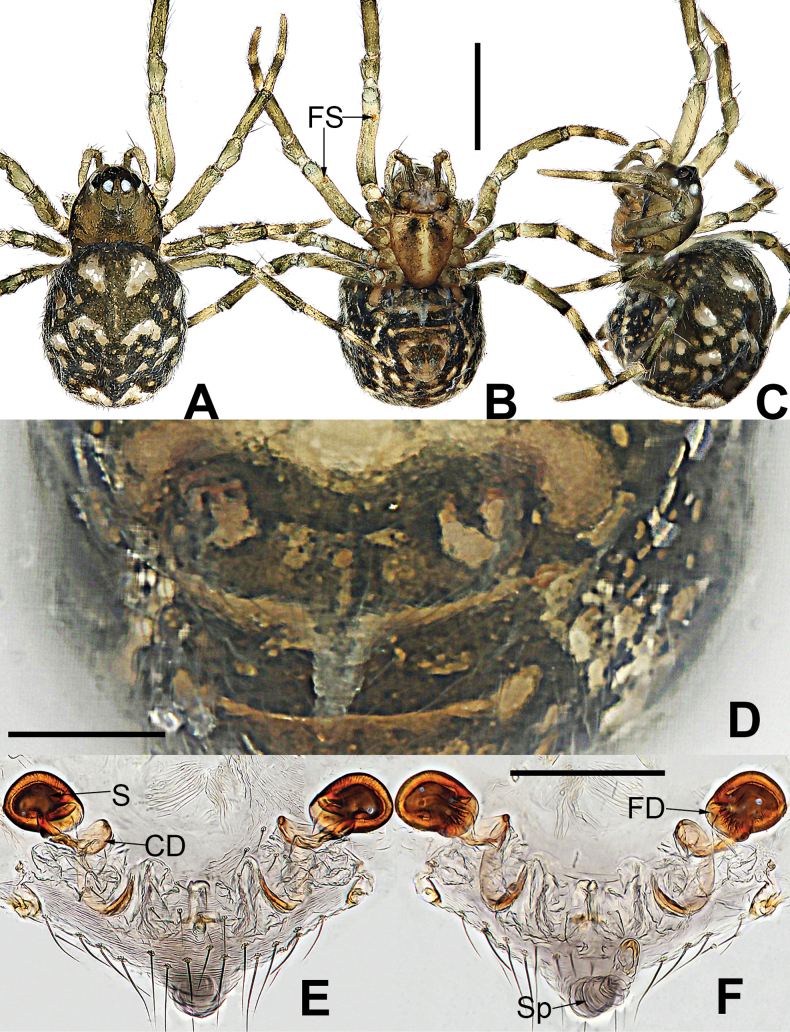
*Microdipoenalisu* sp. nov., female from Yunnan of China **A** habitus, dorsal **B** habitus, ventral **C** habitus, lateral **D** epigyne, ventral **E** vulva, ventral **F** vulva, dorsal. Abbreviations: CD = copulatory duct, FD = fertilization duct, S = spermathecal, Sp = Scape. Scale bars: 0.50 mm (**A–C**); 0.10 mm (**D–F**).

The new species seems similar to *M.jobi* and *M.pseudojobi* in the configuration of the vulva and having a long, soft, membranous scape, but can be distinguished by the subtle difference in the shape of the spermathecae, the ca 3× spacing of the spermathecae, and the copulatory duct enters spermatheca from the posterolateral position (cf. Figs 14E, F, 13B, C and Lin and Li 2008: fig. 12F).

##### Description.

**Female (holotype)**: Total length 1.16. Carapace 0.48 long, 0.44 wide, 0.42 high. Clypeus 0.08 high. Sternum 0.24 long, 0.20 wide. Abdomen 0.68 long, 0.54 wide, 0.60 high. Length of legs: I 1.30 (0.46, 0.18, 0.21, 0.17, 0.28); II 1.26 (0.32, 0.18, 0.24, 0.23, 0.29); III 0.80 (0.20, 0.15, 0.18, 0.16, 0.21); IV 1.24 (0.29, 0.15, 0.28, 0.22, 0.30).

##### Somatic features

(Fig. 14A–C): Body fulvous. Abdomen with irregular paler patches of all sizes. A bright yellow longitudinal stripe at middle line of sternum. Legs pale yellow, with darkish pigment. Carapace pear-shaped. Ocular base black. Eight eyes in two rows. AME black, the rest white. AER procurved, PER straight. Lateral eyes adjacent. Mouthparts pale brown. Labium fused to sternum. Sternum heart-shaped, longer than wide. Femoral spot on legs I and II. Abdomen dorsally short, ovate, posterior integument slightly ridged.

***Epigyne*** (Fig. 14D–F): epigynal area dark, inner structures not visible. Scape long, membranous, slender, ridged, with tiny notch at tip. Most of copulatory ducts membranous, rugose, with weakly sclerotized duct distally, entering from ventral side of spermathecae. Sclerotized, ovoid spermathecae separated by ca 3× their width. Fertilization ducts translucent, membranous, arising from posteromedial side of spermathecae, distally intertwined with membranous parts of copulatory ducts.

**Male.** Unknown.

##### Distribution.

China (Gongshan Co., Yunnan).

#### ﻿ Microdipoena menglunensis

Taxon classificationAnimaliaAraneaeMysmenidae

(Lin & Li, 2008)

0FFA1412-3BCE-5979-B471-2668747983C9

Mysmenella menglunensis Lin & Li, 2008: 506, fig. 13A–I (♂).
Microdipoena menglunensis : Zhang et al. 2022: 71, figs 7A–F, 8A–F, 9A–D (♂♀).

##### Material examined.

3♂ 5♀ (MNHSU-BN110), **China**: Yunnan, Mengla, Menglun, XTBG, Rubber-Tea plantation (about 20 yr.) (21°54.684'N, 101°16.319'E; 569±11 m elev.), by pitfall trapping, 16–31.V.2007, G. Zheng leg.; 3♂ 3♀ (MNHSU-BN111), **China**: Yunnan, Mengla, Menglun, XTBG, Rubber plantation (about 20 yr.) (21°54.483'N, 101°15.978'E; 586±9 m elev.), by searching, 4–11.IV.2007, G. Zheng leg.; 2♂ juv. 5♀ (NHMSU-XSBN20), **China**: Yunnan, Mengla, Menglun, XTBG, tropical rainforest (21°55.020'N, 101°16.500'E; 558 m elev.), 5.X.2017, Y. Lin and Y. Li leg.

##### Diagnosis and description.

See Zhang et al. 2022: 71–75.

##### Distribution.

China (Xishuangbanna of Yunnan).

#### ﻿ Microdipoena shenyang sp. nov.

Taxon classificationAnimaliaAraneaeMysmenidae

7A6E4C31-F5DC-56C1-A2D4-DE1CF189891C

https://zoobank.org/6EB3FEB4-7886-4EBA-B8F2-021EF7883C05

Figs 15 , 16 , 17

##### Type material.

***Holotype*** ♀ and ***paratype*** 7♂ 1♀ (NHMSU-LN01); 3♂ (NHMSU-No.63); 7♂ 6♀ (NHMSU-No.64) **China**: Liaoning Province, Shenyang City, Huanggu District, Beiling Forest Park (42°01.503'N, 123°43.823'E; 352 m elev.), 15–25.X.2010, X. Sun leg.

##### Etymology.

The specific name is derived from the type locality; noun in apposition.

##### Diagnosis.

Male of this new species differs from other congeners by the embolic end twisted into a complex structure, having a cymbial tooth, which is located on the dorsal edge of cymbium, the peculiar shape conductor with a small membranous process and a large, thickened process on lower sides (cf. Figs 12B, C, F, 16A, B, F, 20C, 22B, D, and Lopardo and Hormiga 2015: fig. 132A–F). Female seems similar to *M.jobi* and *M.lisu* sp. nov. in the configuration of vulva and having a long scape with wide at basally and weakly sclerotized at distally, but can be distinguished by the smaller spermathecae spaced by ca 3.5× their diameter and the more visible fertilization ducts (cf. Figs 17C, 13C, 14F).

##### Description.

**Male**: Total length 1.02. Carapace 0.40 long, 0.48 wide, 0.48 high. Clypeus 0.08 high. Sternum 0.28 long, 0.34 wide. Abdomen 0.62 long, 0.62 wide, 0.70 high. Length of legs: I 1.24 (0.30, 0.12, 0.28, 0.26, 0.28); II 1.14 (0.26, 0.12, 0.26, 0.24, 0.26); III 1.03 (0.26, 0.12, 0.22, 0.19, 0.24); IV 1.06 (0.28, 0.12, 0.20, 0.22, 0.24).

##### Somatic characters

**Figure 15.**
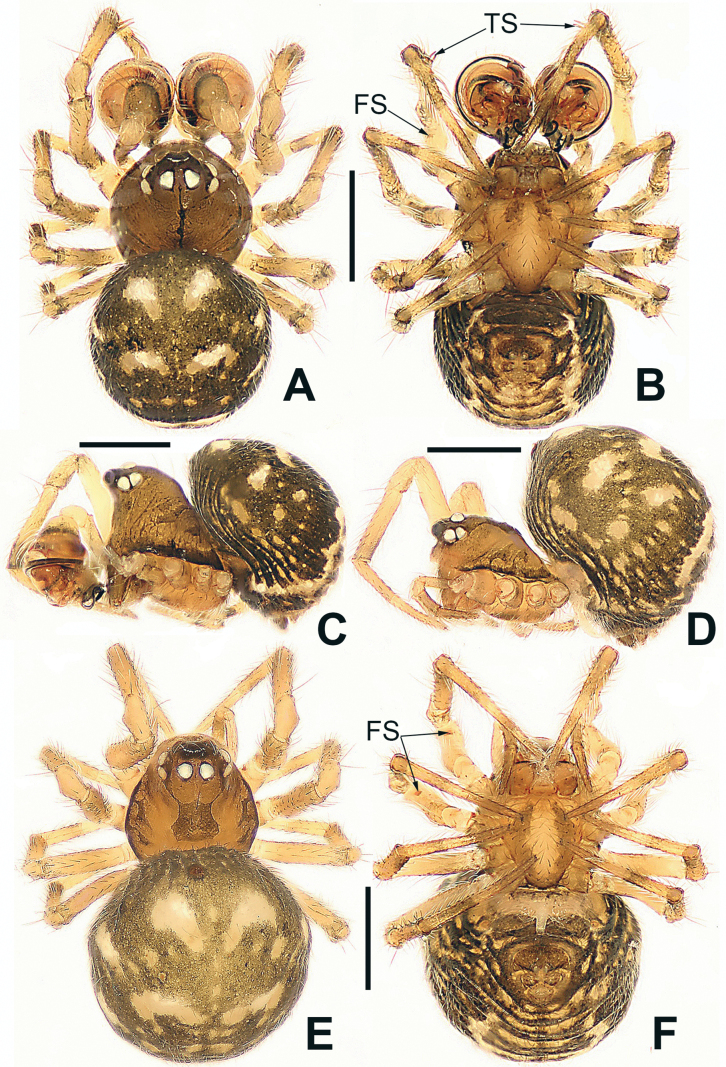
*Microdipoenashenyang* sp. nov., male **(A–C)** and female **(D–F)** from Liaoning of China **A** habitus, dorsal **B** habitus, ventral **C** habitus, lateral **D** habitus, lateral **E** habitus, dorsal **F** habitus, ventral. Abbreviations: FS = femoral spot, TS = tibial spine on male leg I. Scale bars: 0.50 mm.

**(Fig. 15A–C). *Coloration***: carapace dark brown. Chelicera, endites, labium dark yellow, sternum yellow with two small brown spots. Legs yellow-brown. Abdomen black with symmetrical white spots dorsally and ventrally. ***Prosoma***: carapace nearly round in dorsal view and peak-shaped in lateral view. Cephalic part elevated and flat. Sternum scutiform, plump, covered with sparse setae. ***Legs***: covered with setae. Mating clasper on metatarsus I, two strong spines on tibia I. ***Abdomen***: nearly globose in dorsal view, cover pale setae.

**Figure 16.**
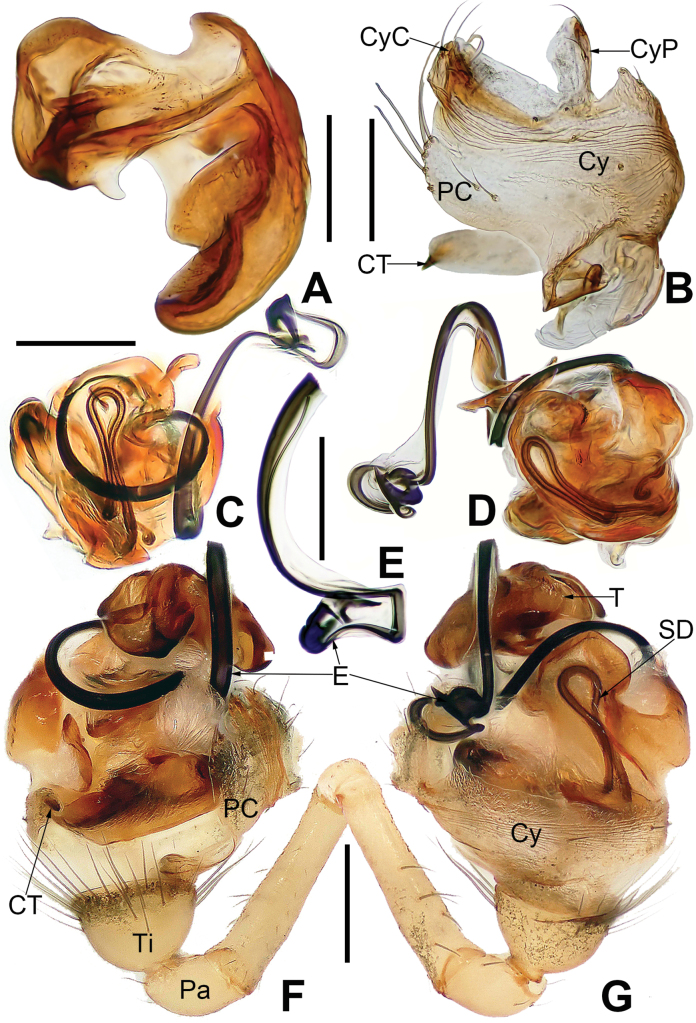
*Microdipoenashenyang* sp. nov. from Liaoning of China **A** conductor, dorsal **B** cymbium, ventral **C** bulbus with conductor removed, prolateral **D** bulbus with conductor removed, retrolateral **E** embolic end, apical-lateral **F** male palp, prolateral **G** male palp retrolateral. Abbreviations: CT = cymbial tooth, Cy = cymbium, CyC = cymbial conductor, CyP = cymbial process, E = embolus, Pa = patella, PC = paracymbium, SD = spermatic duct, T = tegulum, Ti = tibia. Scale bars: 0.10 mm (**A, B, E–G**); 0.20 mm (**C, D**).

***Palp*** (Fig. 16A–G): The cymbium long and translucent, covering 3/4 of the bulb horizontally, cymbial tooth on the distal cymbium, the tip specialized as cymbial conductor, and the other tip forming a large cymbial process. Paracymbium large, with long setae. Conductor 7-shaped, sclerotized, with two large apophyses apically and a tooth-shaped apophysis basally. Tegulum translucent, slightly swollen. Embolus filiform, the tip with complex structure, the other structure coiled into two circles. Spermatic ducts can be seen through translucent tegulum.

**Female (holotype).** Total length 1.25. Carapace 0.42 long, 0.46 wide, 0.42 high. Clypeus 0.08 high. Sternum 0.32 long, 0.28 wide. Abdomen 0.83 long, 0.83 wide, 0.96 high. Length of legs: I 1.20 (0.28, 0.12, 0.24, 0.26, 0.30); II 1.08 (0.26, 0.12, 0.20, 0.24, 0.26); III 1.04 (0.28, 0.12, 0.18, 0.22, 0.24); IV 1.00 (0.28, 0.12, 0.16, 0.20, 0.24).

##### Somatic characters

**(Fig. 15D–F). *Coloration***: carapace pale brown. Ocular base black. Chelicera, endites, labium yellow; sternum yellow with two brown stripes. Legs yellow-brown. Abdomen silvery brown with multiple symmetrical yellow spots dorsally, black with multiple arched yellow stripes and spots ventrally. ***Prosoma***: carapace nearly pear-shaped in dorsal view. Cephalic part slightly elevated. Sternum scutiform, slightly plump, covered in sparse setae. ***Legs***: covered with setae and bristles. Femurs I and II with sclerotized femoral spot. ***Abdomen***: nearly globose in dorsal view, covered with pale setae.

**Figure 17.**
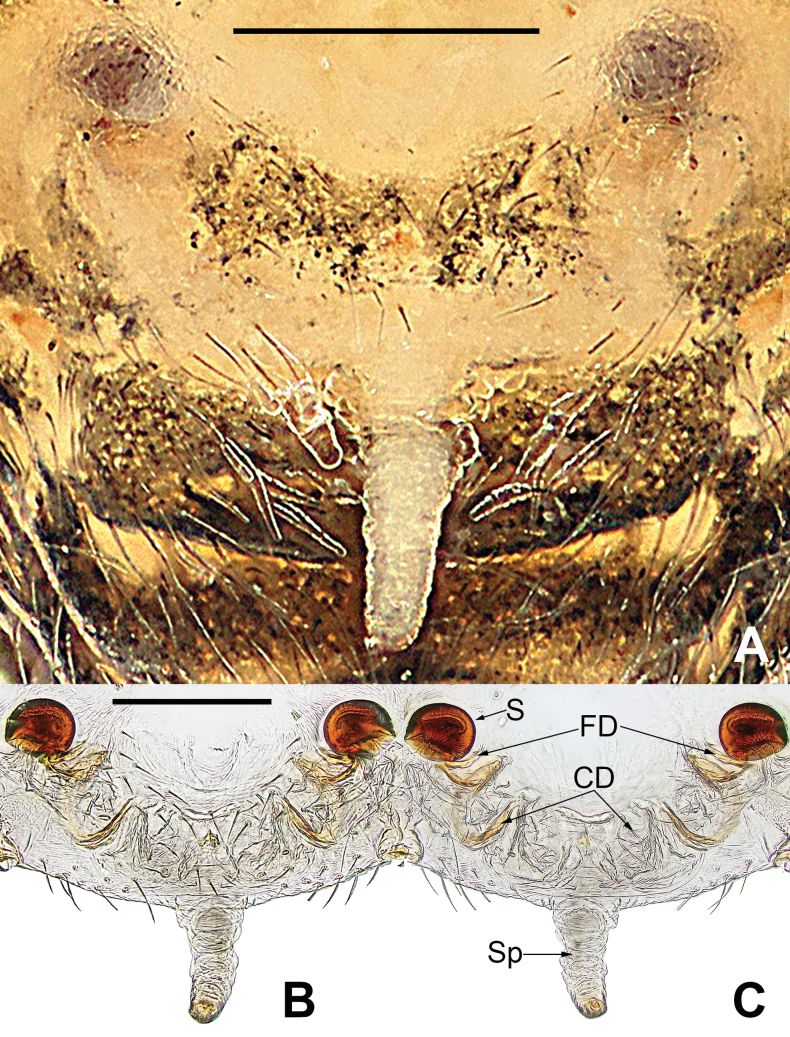
*Microdipoenashenyang* sp. nov. from Liaoning of China **A** epigyne, ventral **B** vulva, ventral **C** vulva, dorsal. Abbreviations: CD = copulatory duct, FD = fertilization duct, S = spermathecal, Sp = scape. Scale bars: 0.10 mm.

***Epigyne*** (Fig. 17A–C): scape long, with thin folds, tip sclerotized. Copulatory duct short, the sclerotized part coiled in two circles, membranous part coiled under the spermathecae. Paired spermathecae semicircular, separated by 4× diameter. Fertilization ducts slightly sclerotized, originated from the lower edge of the spermathecae and bent anteriorly.

##### Distribution.

China (Liaoning)

#### ﻿ Microdipoena thatitou sp. nov.

Taxon classificationAnimaliaAraneaeMysmenidae

C0D0E869-EECE-5484-8622-18B3F60964B7

https://zoobank.org/2FDF9219-4F93-4C86-9D58-672A52EEDC1D

Fig. 18

##### Type material.

***Holotype*** ♀ (NHMSU-No.83) **Laos**: Champasak Province, Muang Bachieng, Ban Lak 35, That Itou (15°11.628'N, 106°06.105'E; 810 m elev.), 26.VI.2009, PW. Jäger and S. Bayer leg.

##### Etymology.

The specific name is derived from the type locality; noun in apposition.

##### Diagnosis.

**Figure 18.**
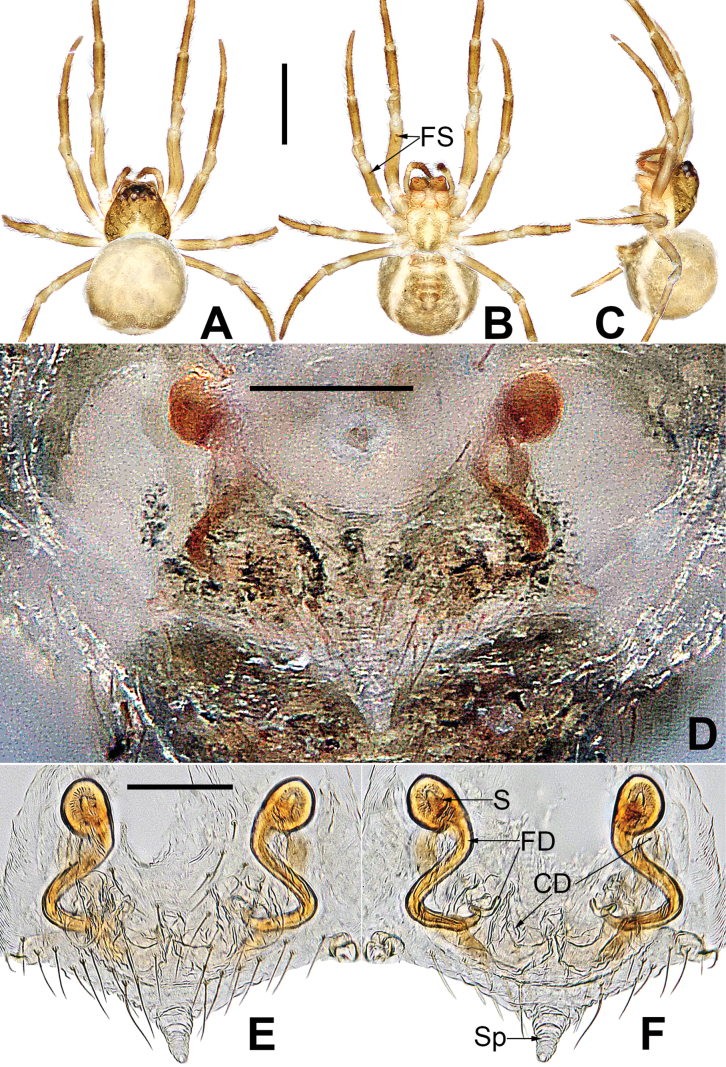
*Microdipoenathatitou* sp. nov., female from Laos **A** habitus, dorsal **B** habitus, ventral **C** habitus, lateral **D** epigyne, ventral **E** vulva, ventral **F** vulva, dorsal. Abbreviations: CD= copulatory duct, FD = fertilization duct, S = spermathecal, Sp = Scape. Scale bars: 0.50 mm (**A–C**), 0.10 mm (**D–F**).

This new species can be distinguished from other congeners by the thickened, long S-shaped fertilization ducts and the entire membranous scape including distal end (Fig. 18D–F).

##### Description.

**Female (holotype)**: Total length 0.82. Carapace 0.24 long, 0.32 wide, 0.24 high. Clypeus 0.06 high. Sternum 0.23 long, 0.18 wide. Abdomen 0.58 long, 0.58 wide, 0.51 high. Length of legs: I 1.13 (0.32, 0.12, 0.23, 0.22, 0.24); II 1.22 (0.24, 0.12, 0.20, 0.18, 0.20); III 0.62 (0.20, 0.08, 0.14, 0.10, 0.10); IV 0.84 (0.24, 0.12, 0.18, 0.14, 0.16).

##### Somatic characters

**(Fig. 18A–C). *Coloration***: carapace yellow centrally, yellow-brown marginally. Ocular base black. Chelicera, endites yellow, labium yellow, sternum pale yellow with two symmetrical black stripes. Legs yellow-brown. Abdomen silvery yellow dorsally, black with symmetrical white and yellow spots, silvery brown with two symmetrical silvery yellow stripes ventrally. ***Prosoma***: carapace nearly pear-shaped in dorsal view. Cephalic part unelevated. Sternum scutiform, covered with sparse setae. ***Legs***: covered with setae and bristles. Femurs I and II with sclerotized femoral spot. ***Abdomen***: nearly spherical, covered with sparse pale setae.

***Epigyne*** (Fig. 18D–F): the structure can be seen through the cuticle. Scape membranous, soft, without weakly sclerotized distal end. Copulatory duct membranous, coiled under the spermathecae. Paired spermathecae oval. Fertilization ducts long, thick, and sclerotized, originated from the lower edge of the spermathecae, the composite structure of fertilization ducts and spermathecae S-shaped.

**Male.** Unknown.

##### Distribution.

Laos (Champasak)

#### ﻿ Microdipoena yinae

Taxon classificationAnimaliaAraneaeMysmenidae

(Lin & Li, 2013)

54E3BF25-49F5-5C98-ACF9-EBAD943BE2EF

Figs 19 , 20

Mysmenella yinae Lin & Li, 2013: 470.
Microdipoena yinae : Lopardo and Hormiga 2015: 783.

##### Type material.

***Holotype***: ♂ (NHMSU) and ***Paratypes*** 14♂ 65♀ (NHMSU), **China**: Sichuan Province, Jiuzhaigou County, Dalu Town, the moss under the forest shrub in the side of Heishui River (33°33.966'N, 103°40.243'E; 2495 m elev.), 28.VI.2011, Y. Lin leg.; 6♂ 31♀ (NHMSU), **China**: Sichuan Province, Jiuzhaigou County, Dalu Town, the forest shrub, at a fork in the road of Dalu Town and Zoige County (33°34.237'N, 103°40.166'E; 2462 m elev.), 28.VI.2011, Y. Lin leg. Examined.

##### Diagnosis.

See diagnosis for *M.gongi* and *M.huisun* sp. nov.

##### Description.

**Figure 19.**
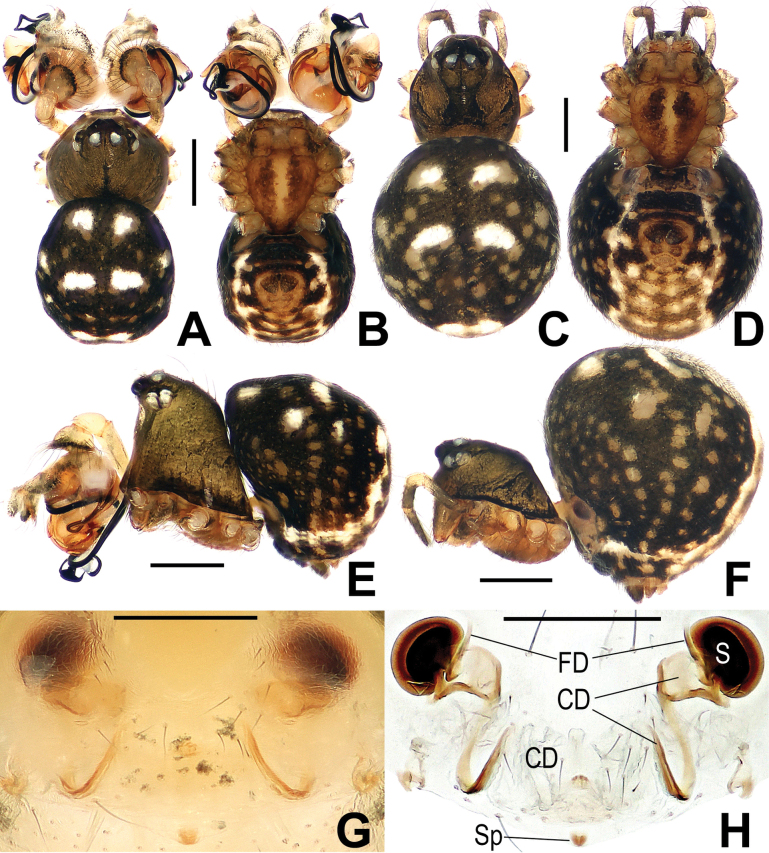
*Microdipoenayinae* Lin & Li, 2013, from Sichuan of China **A** male habitus, dorsal **B** male habitus, ventral **C** male habitus, lateral **D** female habitus, dorsal **E** female habitus, ventral **F** female habitus, lateral **G** epigyne, ventral **H** vulva, dorsal. CD = copulatory duct, FD = fertilization duct, S = spermathecal, Sp = scape. Scale bars: 0.20 mm.

**Figure 20.**
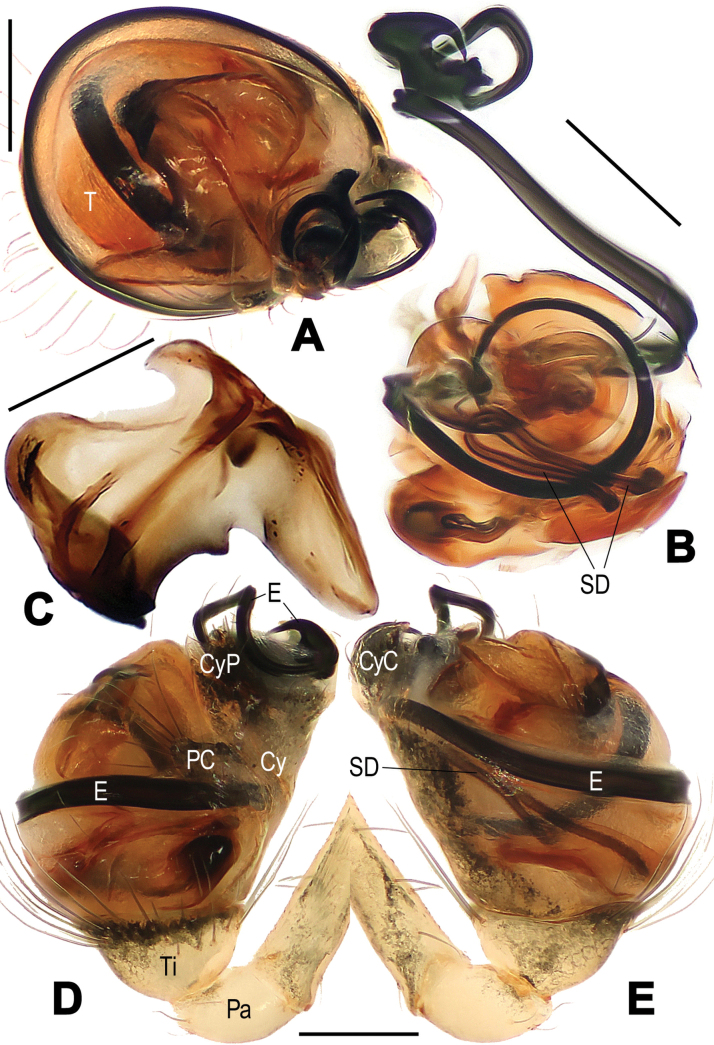
*Microdipoenayinae* Lin & Li, 2013, from Sichuan of China **A** male palp, apical **B** bulbus with conductor removed, dorsolateral **C** conductor, dorsal **D** male palp, prolateral **E** male palp, retrolateral. Abbreviations: Cy = cymbium, CyC = cymbial conductor, CyP = cymbial process, E = embolus, Pa = patella, PC = paracymbium, SD = spermatic duct, T = tegulum, Ti = tibia. Scale bars: 0.10 mm.

See Figs 19, 20 and Lin et al. 2013: 470.

##### Distribution.

China (Sichuan).

#### ﻿ Microdipoena zhulin sp. nov.

Taxon classificationAnimaliaAraneaeMysmenidae

23455E39-FB94-503A-A548-1F76C2703B7A

https://zoobank.org/93F9A4B5-4500-4522-A02C-27433E8D3F84

Figs 21 , 22 , 23

##### Type material.

***Holotype*** ♀ and ***paratype*** 1♂ 3♀ (NHMSU-GX02) **China**: Guangxi Zhuang Autonomous Region, Guilin City, Lingui County, Ertang Township, Yanmendi Village, bamboo forest (25°12.892'N, 100°12.204'E; 165 m elev.), 19.VII.2013, H. Zhao leg.

##### Etymology.

The specific name is derived from the Chinese pinyin for bamboo forest (zhú lín), refers to this species living in this habitats; noun in apposition.

##### Diagnosis.

This new species can be distinguished from other congeners by a combination of the following features of the copulatory organ: having a cymbial tooth near the distal edge of cymbium, the conductor with a small, thumb-shaped, upper process and a right, broad, lower process, the spherical spermathecae separated by ca 2.6× their diameter, the scape of uniform width from base to end, the vulva with two smooth, transparent membranes (probably part of copulatory ducts) (Figs 22B, D, 23D).

##### Description.

**Male**: Total length 1.27. Carapace 0.31 long, 0.44 wide, 0.40 high. Clypeus 0.06 high. Sternum 0.31 long, 0.24 wide. Abdomen 0.96 long, 0.87 wide, 0.93 high. Length of legs: I 0.87 (0.26, 0.10, 0.23, 0.15, 0.13); II 0.64 (0.10, 0.08, 0.18, 0.13, 0.15); III 0.49 (0.18, 0.05, 0.10, 0.08, 0.08); IV 0.64 (0.18, 0.08, 0.18, 0.1, 0.10).

##### Somatic characters

**Figure 21.**
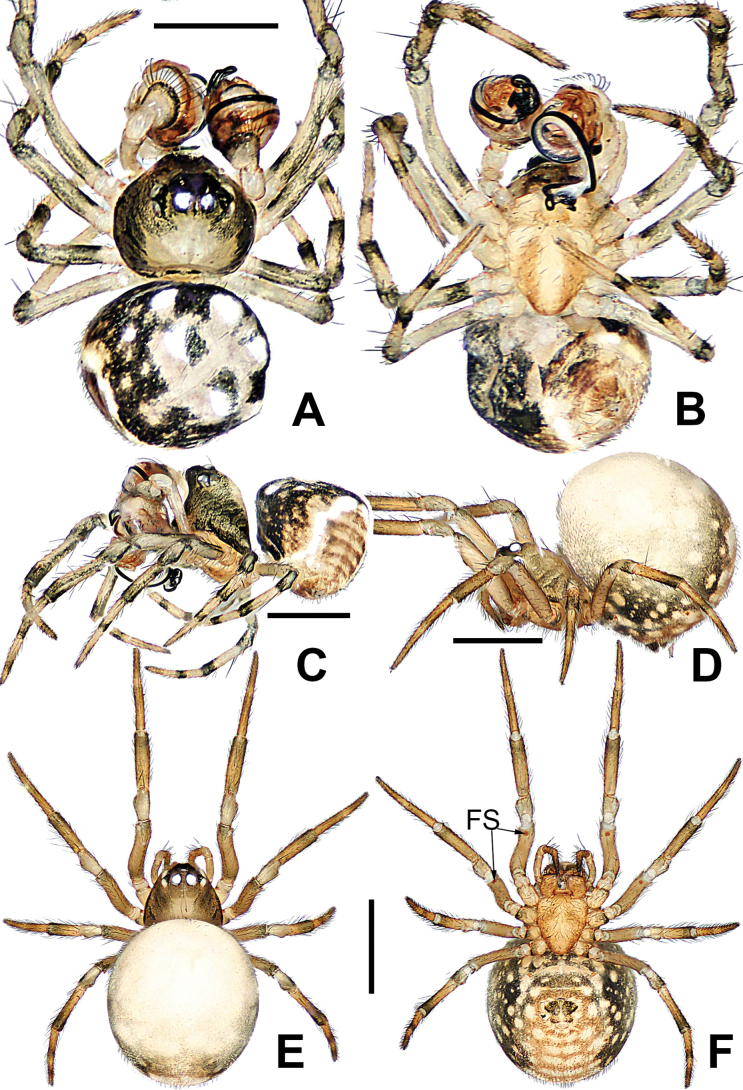
*Microdipoenazhulin* sp. nov., male **(A, B)** and female **(D–F)** from Guangxi of China **A** habitus, dorsal **B** habitus, ventral **C** habitus, lateral **D** habitus, lateral **E** habitus, dorsal **F** habitus, ventral. Abbr.: FS = femoral spot. Scale bars: 0.50 mm.

**(Fig. 21A–C). *Coloration***: carapace silvery yellow centrally, black marginally. Chelicera, endites, labium yellow; sternum yellow with two orange stripes. Legs yellow-black. Abdomen black with large white spots dorsally, yellow with black and white spots ventrally. ***Prosoma***: carapace nearly hexagonal in dorsal view and peak-shaped in lateral view. Cephalic part elevated and flat. Sternum scutiform, plump, covered with sparse setae. ***Legs***: covered with setae. Mating clasper on metatarsus I, two strong spines on tibia I. ***Abdomen***: nearly globose in dorsal view, covered in black setae.

**Figure 22.**
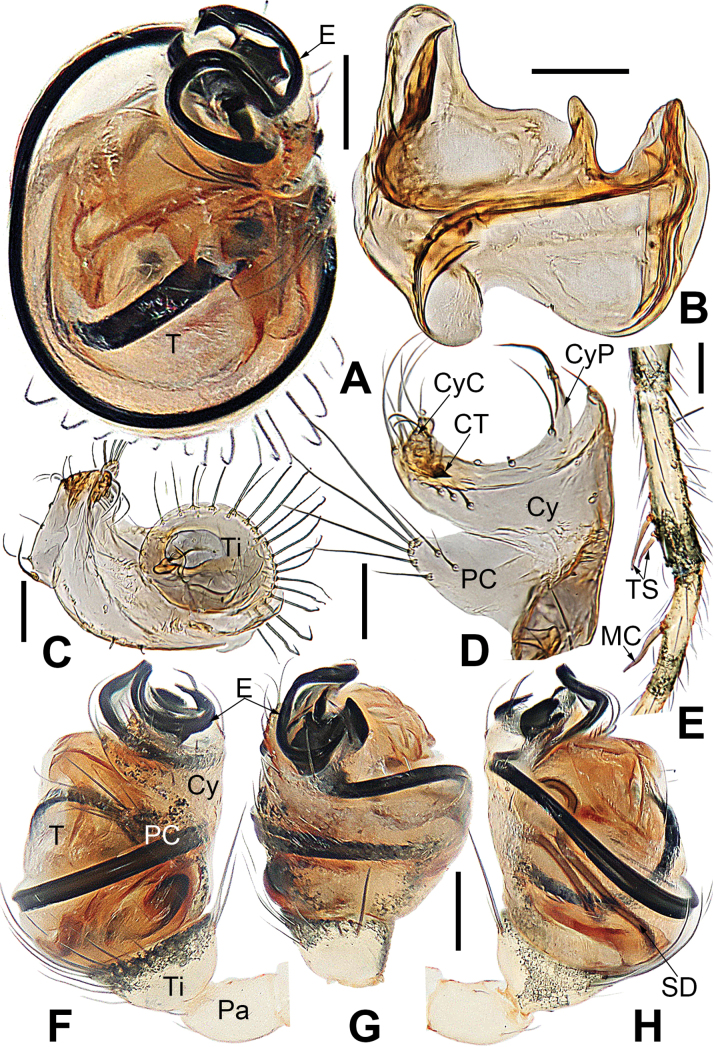
*Microdipoenazhulin* sp. nov. from Guangxi of China **A** palp, apical **B** conductor, dorsal **C** cymbium, apical **D** cymbium, prolateral **E** left tibia I and metatarsus I, prolateral **F** palp, prolateral **G** palp, ventral **H** palp, retrolateral. Abbreviations: CT = cymbial tooth, Cy = cymbium, CyC = cymbial conductor, CyP = cymbial process, E = embolus, MC = mating clasper, Pa = patella, PC = paracymbium, T = tegulum, Ti = tibia, TS = tibial spine. Scale bars: 0.10 mm.

***Palp*** (Fig. 22A, F–H): Cymbium translucent, the tip end of specialized as cymbial conductor, and the other end forming a large cymbial process, a small, sclerotized, cymbial tooth on the outward side of cymbial conductor. Paracymbium finger-shaped, with long setae. Conductor slightly sclerotized, with three large apophyses apically and an arched apophysis basally. Tegulum translucent, slightly swollen. Embolus long, coiled into two circles, the tip coiled and folded into a complex structure. Spermatic ducts can be seen through translucent tegulum.

**Female (holotype).** Total length 1.58. Carapace 0.65 long, 0.75 wide, 0.62 high. Clypeus 0.12 high. Sternum 0.47 long, 0.47 wide. Abdomen 0.93 long, 0.84 wide, 0.92 high. Length of legs: I 1.61 (0.64, 0.18, 0.26, 0.20, 0.33); II 1.39 (0.49, 0.18, 0.26, 0.18, 0.28); III 0.86 (0.28, 0.10, 0.18, 0.14, 0.16); IV 1.04 (0.36, 0.10, 0.18, 0.18, 0.22).

##### Somatic characters

**(Fig. 21D–F). *Coloration***: carapace pale yellow centrally, brown marginally. Ocular base black. Chelicera, endites, labium, and sternum yellow. Legs yellow-brown. Abdomen nearly white dorsally, black with multiple white and yellow spots ventrally. ***Prosoma***: carapace nearly pear-shaped in dorsal view. Cephalic part slightly elevated. Sternum scutiform, slightly plump, covered in sparse setae. ***Legs***: covered with setae and bristles. Femurs I and II with sclerotized femoral spot. ***Abdomen***: nearly globose in dorsal view, covered with black setae.

**Figure 23.**
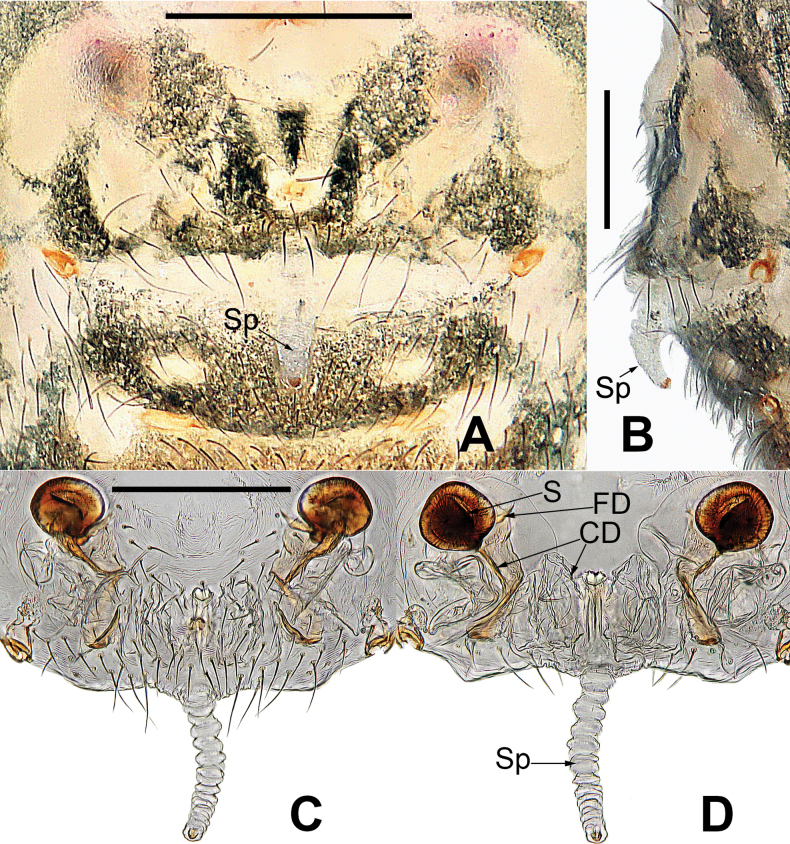
*Microdipoenazhulin* sp. nov. from Guangxi of China **A** epigyne, ventral **B** epigyne, lateral **C** vulva, ventral **D** vulva, dorsal. Abbreviations: CD = copulatory duct, FD = fertilization duct, S = spermathecal, Sp = scape. Scale bars: 0.10 mm.

***Epigyne*** (Fig. 23A–D): scape long, with wide folds, tip sclerotized. Copulatory duct membranous, coiled under the spermathecae. Fertilization ducts slightly sclerotized, originating from the ventral side of the epigyne and bent anteriorly. Paired spermathecae nearly round, separated by nearly double their diameter.

##### Distribution.

China (Guangxi).

## ﻿Discussion

In this paper, we describe a group of species of the genus *Microdipoena* that are mainly native to Eurasia. The morphological characteristics of copulatory organs were compared between multiple congeneric species. Some of the diagnostic features they shared were verified, and can be distinguished from those of other genera (cf. male with two or three tibial spines on the leg I; male palp with a paracymbium, distal part of the embolus coiled and distorted into a complex structure in most species; Lopardo and Hormiga 2015). To test whether our taxonomic decisions and their classification status are correct, we also conducted phylogenetic analyses based on molecular evidence for ten named and five undescribed *Microdipoena* species. Our phylogenetic analysis shows that the monophyly of this genus is valid and these taxonomic judgments proposed by us in this study are correct. However, the male characters of copulatory organs of three species are unknown due to inadequate sampling (*M.huisun* sp. nov., *M.lisu* sp. nov., and *M.thatitou* sp. nov.).

According to the reported distribution records, the genus is mainly distributed in the continents of Asia and Africa and nearby islands. Most species of the genus are endemic, some of which have multiple distribution sites (*M.elsae*, *M.nyungwe*, *M.samoensis*), and a few may have expanded distribution ranges as a result of introduction (*M.guttata* and *M.jobi*). The origin and diffusion history of *Microdipoena* are questions worthy of further discussion. Faunal surveys and diversity studies of this genus are a prerequisite for answering these questions, but much work remains to be done.

## Supplementary Material

XML Treatment for Microdipoena

XML Treatment for Microdipoena elsae

XML Treatment for Microdipoena gongi

XML Treatment for Microdipoena huisun

XML Treatment for Microdipoena jobi

XML Treatment for Microdipoena lisu

XML Treatment for Microdipoena menglunensis

XML Treatment for Microdipoena shenyang

XML Treatment for Microdipoena thatitou

XML Treatment for Microdipoena yinae

XML Treatment for Microdipoena zhulin

## ﻿Additional information

### Author ORCIDs

Yucheng Lin https://orcid.org/0000-0002-5054-0633

### Data availability

All of the data that support the findings of this study are available in the main text.
